# Social validity as an interactive process

**DOI:** 10.1002/jaba.70079

**Published:** 2026-08-14

**Authors:** Toni Rose T. Agana, Tina M. Sidener, Heather M. Pane, Onada Pauline Macaraeg, Nicole M. Rodriguez, Sharon A. Reeve

**Affiliations:** ^1^ Department of Applied Behavior Analysis Caldwell University Caldwell NJ USA; ^2^ Munroe‐Meyer Institute University of Nebraska Medical Center Omaha NE USA; ^3^ Department of Behavioral Science Daemen University Amherst NY USA; ^4^ Between and Beyond Lines Learning Intervention Services Baguio City PH

**Keywords:** acceptability, assessment, interactive process, social validation, social validity

## Abstract

Social validity in applied behavior analysis remains critically important. However, assessing social validity and responding to its results pose challenges for the field. Social validity assessments are often treated as a one‐time measure conducted after intervention. Researchers have urged consideration of social validity as an ongoing, dynamic, and interactive process (i.e., interventions are adjusted based on social validity assessment results). Yet, there has been limited discussion on the interactive process of social validity and the variables that may influence it. This article (a) reviews definitions of social validity and the purpose of its assessment; (b) examines how studies published in the *Journal of Applied Behavior Analysis* from 2010 to 2020 have assessed social validity as an interactive process; and (c) offers considerations for researchers and practitioners to enhance opportunities for an interactive process of social validity, including issues related to the measurement rigor and data interpretability of social validity assessments.

The purpose of the *Journal of Applied Behavior Analysis* (*JABA*), as described in the first issue and by Wolf (1978), was “for the publication of applications of the analysis of behavior to problems of social importance” (p. 203). Wolf (1976, 1978) expanded on this by making a case for subjective measurement and urging behavior analysts to extend their evaluation of interventions beyond objective measures of efficacy and effectiveness. This position was notable given that applied behavior analysis is considered a natural science concerned with the objective measurement of natural events.

Wolf (1976, 1978) recommended that researchers and practitioners examine the *social validity* of an intervention—that is, whether consumers are satisfied with the goals, procedures, and outcomes of behavior‐change efforts. In addition, Kazdin (1977) advocated for the structured and rigorous measurement of social validity, which contributed to efforts to operationalize and assess these constructs using validated assessment methods (e.g., Kazdin, 1980). The contributions of Wolf (1976, 1978) and Kazdin (1977) introduced a framework for incorporating social validity assessments into research and practice. This is especially relevant in light of recent concerns about applied behavior analysis from published (e.g., Ne′eman, 2010; Shkedy et al., 2021) and unpublished sources, underscoring the importance of ensuring that interventions are not only effective but also acceptable and meaningful to stakeholders. However, assessing social validity and responding to social validity assessment results remain challenging areas for the field (Nicolson et al., 2020).

Since the call for social validity (Kazdin, 1977; Wolf, 1976, 1978), the concept has undergone considerable advancements, with researchers developing validated tools for subjective measurements (e.g., Kazdin, 1980; Kelley et al., 1989), defining its parameters (e.g., Schwartz & Baer, 1991), examining its various terminologies (e.g., Huntington et al., 2023), refining methods of assessment (e.g., Ferguson et al., 2019), and discussing its purpose (e.g., Gresham & Lopez, 1996). Although definitions and measurement strategies have advanced considerably, far less attention has been given to how the results of social validity assessments are used as part of decision making across intervention stages. Researchers have noted that social validity assessments are often treated as a one‐time measure, typically conducted once the study has been completed (Leif et al., 2024). Others have proposed that social validity be an ongoing, dynamic, and interactive process (Fawcett, 1991; Finney, 1991). Finney (1991) discussed that doing so can enhance the social validity of interventions and demonstrate the benefits of mutual influence between researcher and consumer.

Despite these considerations (e.g., Finney, 1991), limited discussion and empirical documentation of how often—and in what ways—social validity data are used as part of an interactive process in applied behavior analysis remain. If this gap is not addressed, opportunities for stakeholder or consumer participation that could guide ongoing evaluation and adjustment may be missed and increase risk of implementing interventions that are technically efficacious but socially unacceptable. Overall, although social validity has been conceptualized as a process beyond measurement, there is limited empirical evidence documenting how social validity assessment results have been used to inform intervention adjustments across stages.

Therefore, the purpose of this article is to address an empirical and conceptual gap in the field by examining how social validity assessment can function as an ongoing evaluative mechanism. Specifically, we present a discussion informed by a reanalysis of the findings by Leif et al. (2024), the most recent review of social validity assessments in studies published in *JABA*. We also offer an overview of how the interactive process of social validity can be applied and expanded in research and practice within applied behavior analysis. The article has three primary objectives: (a) review definitions of social validity and the purpose of its assessment; (b) examine how studies published in *JABA* from 2010 to 2020 have assessed social validity as an interactive process; and (c) offer considerations for researchers and practitioners to enhance opportunities for an interactive process of social validity, including issues related to the measurement rigor and data interpretability of social validity assessments. Although social validity and related constructs have been examined in other disciplines (e.g., Sekhon et al., 2017), this article focuses primarily on the conceptualization and application within applied behavior analysis.

## WHAT IS SOCIAL VALIDITY?

According to Wolf (1976, 1978), social validity involves evaluating the significance of intervention goals, the acceptability of procedures, and the meaningfulness of outcomes. He further noted that assessing social validity depends on subjective information, such as the opinions and judgments of others. Although Wolf defined social validity and its importance, he provided relatively little guidance regarding how these judgments should be assessed. In response to Wolf (1976), Kazdin (1977) advocated for using validated measures to assess the clinical significance or social validity of behavior change. This led to the development of validated measures of social validity in applied behavior analysis, including the Treatment Evaluation Inventory (Kazdin, 1980) and Treatment Evaluation Inventory–Short Form (Kelley et al., 1989).

Additionally, Schwartz and Baer (1991) expanded on Wolf's conceptualization by highlighting that if interventions (a) lack meaningful goals, (b) are delivered in unacceptable ways, or (c) fail to produce valued outcomes, then consumers may discontinue, avoid, or oppose them, which they called social *invalidity*. They recommended that social invalidity should also be assessed. Schwartz and Baer argued that dissatisfied consumers should be encouraged to complain and do so early so that researchers and practitioners can respond to social validity assessments. They also addressed concerns that reliance on subjective measures of social validity could weaken the field's emphasis on objective data. Instead, Schwartz and Baer emphasized that social validity is intended to complement, not replace, objective measures.

Kazdin (1977) was among the first to classify social validity assessments into two types: (1) normative (or social) comparisons, such as comparing outcomes to typical or desired behavior, and (2) subjective evaluations, such as ratings of perceived acceptability provided by relevant stakeholders (e.g., participants, caregivers, teachers). However, he also highlighted limitations in the interpretation of these measures. For example, identifying an appropriate normative comparison may be difficult. Furthermore, the selected comparison group may provide unrealistic standards for evaluation due to diverse differences in background (e.g., education, socioeconomic status, demographic characteristics). Similarly, subjective evaluations raise important questions regarding measurement rigor and interpretability. Subjective evaluations may also exhibit low correspondence with observed behavior.

Kennedy (1992) emphasized the need for measures that more sensitively capture behavior changes in social contexts. For example, Likert‐type scales completed by parents or teachers may not reflect the subtle ways an intervention affects behavior in natural settings. A rating to the question “This intervention is an effective way to teach play skills” for a study increasing a child's play skills may not capture whether the child is actively exploring toys, initiating play with peers, or sustaining play engagement over time. Extending earlier direct approaches, such as normative comparisons, researchers (e.g., Common & Lane, 2017; Ferguson et al., 2019) have proposed additional methods for directly evaluating social validity, such as treatment preference evaluations using a concurrent‐chains procedure (Hanley et al., 1997). Despite these recommendations, subjective evaluations remain the most widely used method (Ferguson et al., 2019). Moreover, even as social validity assessment methods have expanded, limited attention has been given to how social validity assessment results should be used.

It is also essential to consider who evaluates it (e.g., consumers) because different perspectives can influence the results of social validity assessments. The selection of evaluators can influence both the information gathered and the interpretation of findings. Schwartz and Baer (1991) differentiated among four types of consumers: (a) direct consumers, (b) indirect consumers, (c) members of the immediate community, and (d) members of the extended community. Direct consumers are individuals receiving services. Indirect consumers are strongly affected by the intervention but are not its direct recipients, such as caregivers and teachers. Members of the immediate community are individuals who regularly interact with direct and indirect consumers through close proximity, such as neighbors or regular bus drivers. Members of the extended community are individuals who may not directly interact with consumers but live in the same community, such as supermarket employees who observe individuals applying newly learned skills. Other researchers (e.g., Kazdin, 1977) also highlighted expert judges, or professionals with knowledge relevant to the treatment context, as an additional valuable source of perspective.

Another important issue in behavioral research is the challenge of labeling and reporting social validity data that can hinder researchers' ability to summarize and evaluate social validity assessments, particularly when terminology is used inconsistently (Huntington et al., 2023). Nicolson et al. (2020) noted that terms such as social significance and social validity are sometimes used interchangeably in the literature; however, they argue that the terms have distinct concepts (i.e., social significance refers broadly to societal acceptability, whereas social validity refers more specifically to the appropriateness and acceptability of interventions). Huntington et al. (2023) found that whereas “social validity” was the most commonly used term across journals, other phrases, such as “acceptability,” were frequently employed to describe the same concept. They suggested that the relatively low reporting of social validity in past research may partly reflect these inconsistent terminologies and recommended that related terms (e.g., acceptability) be considered when reviewing social validity data. Furthermore, identifying social validity assessments in literature reviews can be more challenging when studies include such measures but neither explicitly label them as social validity nor categorize them under a dedicated “Social Validity” section. These challenges may also obscure whether and how researchers have used social validity data to guide intervention implementation.

Thus, the development of social validity has highlighted complexities in definitions and methodologies as well as practical implications for intervention design, delivery, and evaluation. Although advancements in definitions, measurement approaches, evaluator guidance, and terminology have clarified what social validity is, what to measure, who evaluates it, and how it is discussed, there remains limited guidance on how social validity assessment results should inform intervention decisions. This raises an important question for researchers and practitioners: Beyond documenting whether consumers find an intervention acceptable, what is the purpose of social validity measures within applied behavior analysis?

## WHAT IS THE PURPOSE OF SOCIAL VALIDITY MEASURES?

Hawkins (1991) emphasized that consumer satisfaction or social validity measures hold value for several practical reasons, provided they are interpreted cautiously. One potential purpose of social validity measures is to identify when additional education is needed (Hawkins, 1991). For example, if parents respond negatively to implementing a token economy at home (e.g., describing it as “bribing” or expressing concerns about feasibility), this feedback may signal the need to further explore their perspectives and collaboratively determine whether additional clarification of the rationale and benefits is warranted rather than abandoning the procedure. Similarly, Vollmer and Pendergrass (2025) noted that providing clear explanations on the scientific evidence of the procedures before, during, and after implementation not only aligns with ethical standards (Behavior Analyst Certification Board, 2020) but also reflects compassionate care (Rohrer et al., 2021; Rohrer & Weiss, 2023). Although behavior analysts are not expected to convince consumers of scientific facts, it is important that service recipients can understand the rationale, evidence, and efficacy of the procedures being implemented (Vahdat et al., 2014).

Researchers (e.g., Hawkins, 1991) have also underscored that an important function of social validity measures is their capacity to guide procedural adjustments that enhance treatment acceptability. For example, Hawkins (1991) described a foster‐family‐based program in which youth often objected to the use of a point system or a token economy even after extended exposure. Although such responses suggested low acceptability, staff providing the intervention acknowledged the effectiveness of token economies and chose not to eliminate them. Instead, they modified the procedure by allowing youth who performed well to discontinue the system, with the agreement that it would be reinstated if behavior declined. Although the effect of this adjustment was not formally evaluated, Hawkins suggested it may have contributed to improved outcomes. This example illustrates how consumer satisfaction data, even if not taken at face value, can guide adjustments with potentially favorable effects.

Other researchers have also highlighted the role of social validity in guiding interventions. Several frameworks emphasize the importance of interpreting measures carefully and using them to inform practice. Gresham and Lopez (1996) conceptualized social validity as both a product of assessment and driver of the intervention process, arguing that more frequent and systematic social validity assessments could improve their utility for decision making. Similarly, Ferguson et al. (2019) noted that gathering consumer feedback early in the development of an intervention can strengthen the intervention by identifying variables that contribute to social invalidity as well as those valued by consumers. More recently, Leif et al. (2024) discussed how ongoing social validity assessments could allow researchers to adjust skill‐based treatment contexts to incorporate preferred components (e.g., schedule, type of demands), which could increase participation.

**FIGURE 1:**
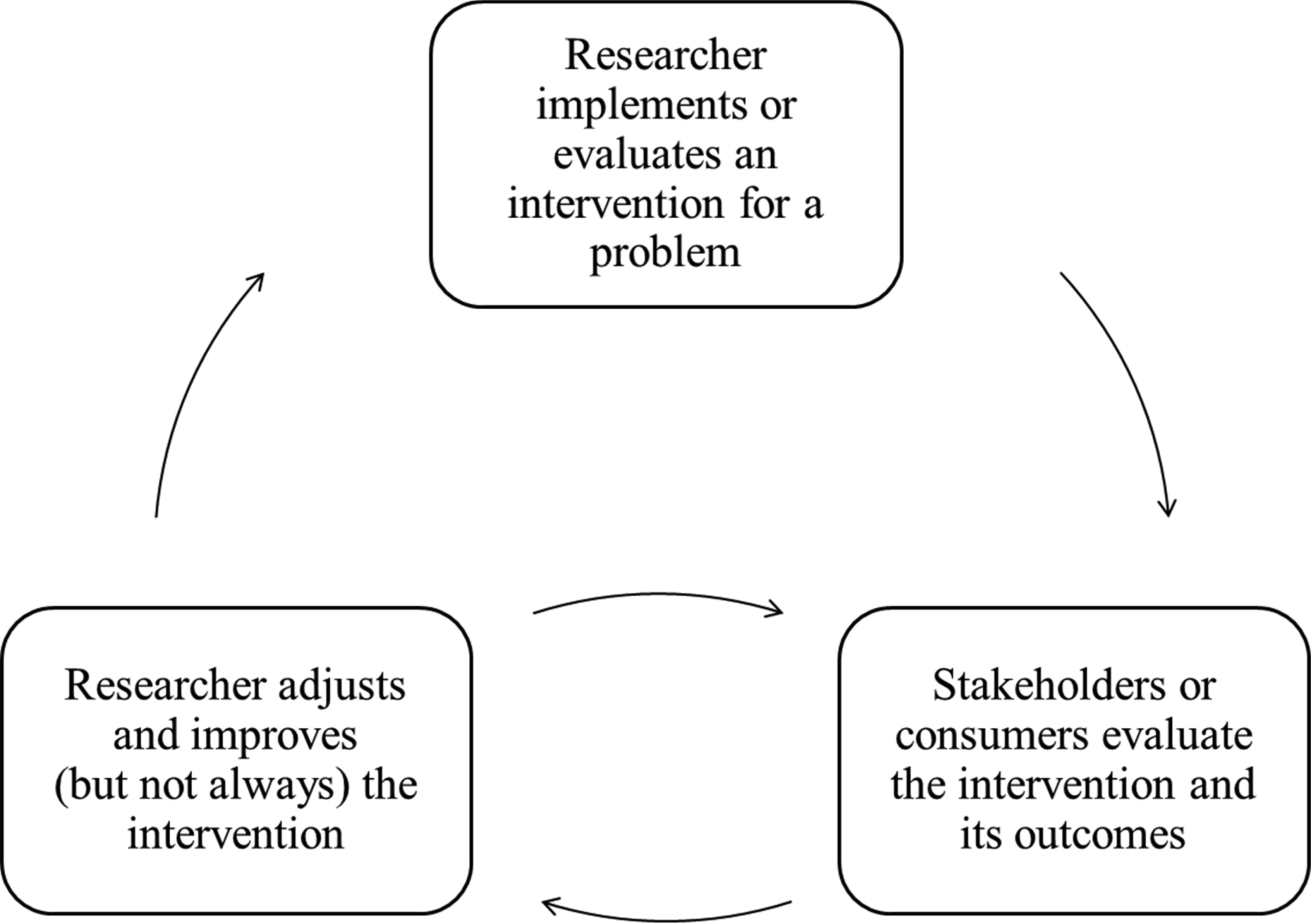
The interactive process of social validation for behavioral research. This diagram is adapted from the description of the interactive process of social validation for behavioral research by Finney (1991).

Finney (1991) argued that social validity assessments are too often treated as a one‐time measure rather than as part of an *interactive process* (i.e., making intervention adjustments based on social validity assessment results). In contrast, they conceptualized social validity as a continuous evaluation of goals, procedures, and outcomes in which researchers adapt the intervention based on results. Figure 1 illustrates this conceptualization of social validity as an interactive process involving continuous evaluation and intervention adaptation. Fawcett (1991) similarly recommended making adjustments when social validity assessments reveal disagreement or dissatisfaction across social validity dimensions (i.e., goals, procedures, outcomes). For example, researchers and practitioners may alter interventions when results show that goals are insignificant, procedures are unacceptable, or outcomes are unimportant. Although others (i.e., Fawcett, 1991; Finney, 1991) emphasized ongoing evaluation and modification, their descriptions may align more closely with an *iterative* process, which is a repeated cycle of assessment and adjustment. In the present article, we conceptualize an interactive process as a bidirectional and mutual influence between consumers and researchers or practitioners to select, adapt, implement, and evaluate behavior‐analytic goals, procedures, and outcomes.

Perhaps the lack of discussion on the interactive process of social validity is partly influenced by the timing of social validity assessments. As reported in the most recent literature review on social validity assessments, Leif et al. (2024) found that assessment is often conducted after intervention, which limits opportunities for responsive changes. For example, when participants provide anonymous feedback after intervention, dissatisfaction may be difficult to address. In academic contexts, graduate students conducting thesis or dissertation projects may also lack the time or motivation to respond after intervention data once the study concludes. Thus, the timing and structure of social validity assessments may play a critical role in whether social validity functions as a static measure or as an ongoing, interactive process. Furthermore, unstructured evaluations such as anecdotal reports may capture intervention adjustments more than structured social validity assessments, yet they are often excluded from reviews of social validity research (Ferguson et al., 2019). Despite the potential of an interactive process when obtaining social validity measures, such applications have not been described or documented in previous literature reviews.

## INTERACTIVE PROCESS OF SOCIAL VALIDITY IN *JABA* (2010–2020)

To better understand how social validity assessment results have been used in the interactive process, we examined the articles included in the most recent review by Leif et al. (2024) on social validity assessments in *JABA*. Our goal was not to replicate their comprehensive search but to build on their included articles to highlight representative examples of social validity measurement, with particular attention to the interactive process when conducting social validity assessments. Through the reanalysis of the findings reported in Leif et al., we sought to present a discussion on identified current practices that reflect or limit interactive processes and inform how social validity can be incorporated into research and practice.

Therefore, we conducted a full‐text review of the articles reported by Leif et al. (2024) by examining whether the results of social validity assessments informed adjustments to interventions. We also searched the literature using the alternative terms for social validity discussed by Huntington et al. (2023).^1^ Consistent with Leif et al., we included *JABA* studies published between 2010 and 2020 that reported qualitative or quantitative social validity data. These social validity data included subjective evaluations (e.g., ratings, judgments, or statements; Kazdin, 1977; Kennedy, 1992; Wolf, 1978) and direct evaluations (e.g., behavioral observations; Common & Lane, 2017), including normative comparisons (i.e., comparing observed behavior with that of others; Kazdin, 1977; Kennedy, 1992) and treatment preference evaluations (e.g., Hanley et al., 1997). The variables we examined extended Leif et al. by also incorporating unstructured subjective evaluations as social validity assessments, such as anecdotal reports that reflect participants' perceptions about treatment (Ferguson et al., 2019). We did not, however, consider best‐practice procedures to qualify as social validity assessments such as conducting a functional analysis or functional behavioral assessment, using function‐based goals or skills assessments, conducting preference assessments to identify reinforcers, specifying inclusion or exclusion criteria, or completing intake evaluations to rule out medical concerns. Table 1 lists the social validity assessment types included. In total, we analyzed 165 studies: the 160 identified by Leif et al. plus five additional articles identified by searching alternative terms for social validity, which met Leif et al.'s criteria but were not included in their review. Supporting Information A provides the full list of articles included in the review.

**TABLE 1:** Social validity assessment types.

Type of social validity assessment	Definition	Example
Subjective evaluation	A social validity assessment that provides reports based on individual's ratings, judgments, or statements indicating social validity (Kazdin, 1977; Kennedy, 1992; Wolf, 1978). This category also includes subjective evaluations based on direct observation of sessions (e.g., watching video recordings). Common measurement formats include Likert‐type rating scales, non‐Likert‐type questionnaires (e.g., multiple‐choice, yes/no, open‐ended questions), structured or semistructured interviews, and observer ratings following in vivo or video‐based observations (cf. Leif et al., 2024).	(See examples below)
1a. Structured: Validated measures	A type of subjective evaluation that uses standardized methods that have undergone psychometric evaluation demonstrative of reliability and validity for measuring a specified construct (Furr, 2022).	The Intervention Rating Profile (IRP; Witt & Martens,^1983^; Witt et al., 1984) was used to assess the acceptability of reinforcement‐based interventions for problem behavior before the start of the intervention (Gabor et al., 2016).
1b. Structured: Unvalidated measures	A type of subjective evaluation that uses standardized methods that have been developed for a specific study or clinical context and have not been subjected to systematic psychometric evaluation.	An author‐developed survey was administered to parents at the end of the study to assess their satisfaction with their child's enjoyment of yoga as a newly acquired skill (Gruber & Poulson, 2016).
1c. Unstructured	A type of subjective evaluation that relies on anecdotal reports of social validity (Ferguson et al., 2019).	To evaluate the use of video self‐evaluation and video feedback to increase the accuracy of yoga poses. Three yoga instructors agreed that the target behavior poses were appropriate for healthy beginning yoga students (Downs et al., 2015).
2 Direct evaluation	A social validity assessment based on observing behavior to measure social validity (Common & Lane, 2017). Common measurement formats include direct observation of behavior, intervention preference or choice measures, and concurrent‐chains preference assessments (cf. Leif et al., 2024).	(See examples below)
2a. Normative comparison	A type of direct evaluation in which an individual's performance is compared with that of individuals with typical or desirable behavior (Common & Lane, 2017; Kazdin, 1977; Kennedy, 1992).	To teach autistic children to tact olfactory stimuli, six typically developing children provided normative data on scent names and categories to ensure age‐appropriate tacts (Dass et al., 2018).
2b. Treatment preference evaluation	A type of direct evaluation that measures an individual's selection or engagement resulting in treatment preference data (Common & Lane, 2017).	When comparing methods to teach foreign‐language targets to young children, researchers assessed preferences for tact training and mixed training (Matter et al., 2020).

*Note*: Common measurement formats are described within the definitions of broader social validity assessment categories to support interpretation by readers unfamiliar with Leif et al. (2024). These formats illustrate specific social validity types that were included in Leif et al.

Coding categories were developed by the first and second authors. Four primary categories were identified to capture key aspects of the interactive process of social validity assessments in research: (1) timing of social validity assessment, (2) adjustment of the intervention, (3) social validity dimension, and (4) manuscript location. The first, third, and fourth authors independently coded the articles. Interrater agreement was calculated as the number of agreements divided by the total agreements plus disagreements. For a subset of 50 of the 165 (30%) included studies, secondary evaluators (i.e., third and fourth authors) independently coded all variables. Agreement was recorded when both raters assigned identical codes for a variable. Across all coded variables, mean interrater agreement was 91.58% (range: 73.68%–100%). Coders independently reviewed all articles and met after initial coding to resolve discrepancies. For each disagreement, coders discussed the rationale and referred to the operational definitions of the variables and coding guidelines. Each disagreement was reevaluated and reached consensus on the most appropriate code based on the definitions and criteria of the coding categories. No changes to previously coded articles were required once consensus procedures were established; therefore, interrater agreement data reflect initial independent coding.

### Timing of the assessments

Similar to Leif et al. (2024), we coded the timing of social validity assessments to provide context for when intervention adjustments were completed. Social validity assessments were categorized by their timing as follows: (a) *before*, when social validity data (i.e., importance of treatment goals, procedure acceptability, or behavior outcomes significance) were obtained prior to implementation of the intervention; (b) *during*, when social validity data were obtained after the intervention began but before it concluded; (c) *after*, when social validity data were obtained once the intervention was completed (Ferguson et al., 2019; Kennedy, 1992; Wolf, 1978); (d) and *unclear*, when social validity data were obtained but timing could not be determined due to insufficient detail (Leif et al., 2024).

**FIGURE 2:**
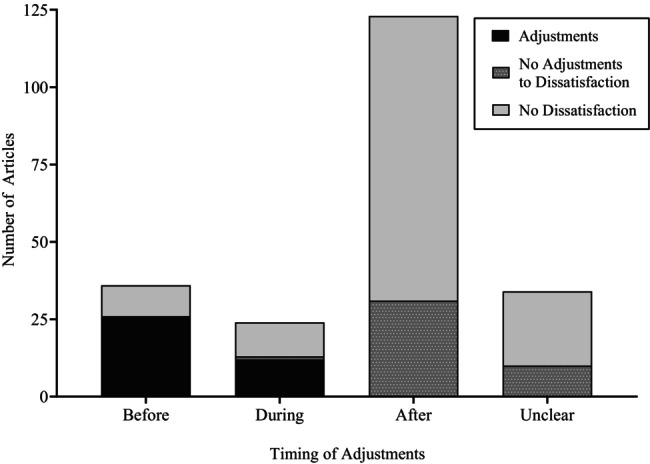
Number of articles reporting adjustments by timing of social validity assessments. Bars represent articles in the review and the timing of social validity assessments (i.e., before, during, after, or unclear). Within each timing category, bars are stacked to show the proportion of articles that reported adjustments, reported no adjustments to dissatisfaction, or reported no dissatisfaction based on social validity assessment results. Total articles within each intervention timing category were as follows: before (*n* = 36), during (*n* = 24), after (*n* = 123), and unclear (*n* = 34).

Although timing was not the primary focus of our study, we coded it as a first step in examining the interactive process to identify when adjustments to interventions from social validity assessment results occurred. Figure 2 (and Supporting Information B) presents the total number of articles that conducted social validity assessments across timing categories. Overall, the distribution of social validity assessment timings reviewed generally aligned with Leif et al. (2024), with some notable differences discussed. First, we included five additional studies not identified by Leif et al. and identified more social validity assessments conducted before intervention. However, it is possible that some conducted social validity assessments before intervention but did not report them. Second, differences in the timing of social validity assessments between our review and Leif et al. may be attributed to our inclusion of unstructured subjective evaluations (e.g., anecdotal reports) and normative comparisons as direct evaluations, which were not specified in their review. For example, in a study on auditory feedback in body weight training (Vorbeck & Bördlein, 2020), participants provided anecdotal reports, viewing their participation as an opportunity to exercise more regularly. Unstructured subjective evaluations may be more common than previously reported, as they have not been consistently included in prior reviews (Ferguson et al., 2019).

Another notable difference between our findings and those of Leif et al. (2024) is the higher number of social validity assessments we identified with unclear timing, possibly due to ambiguity in how the timing of these assessments was described in some studies. Additionally, some discrepancies in interrater agreement during our review were related to social validity assessment timings. It is possible that unclear timing descriptions could be due to the assumption that social validity typically occurs after intervention, leading authors to omit details about when social validity assessment was conducted.

### Adjustments to intervention

After coding the social validity assessments, we examined the interactive process by analyzing how social validity results informed the initial design of interventions or any subsequent adjustments to interventions. We coded three categories based on social validity assessment results: (a) *adjustments*, when results informed the development or modification of an intervention; (b) *no adjustments to dissatisfaction*, when results indicated unfavorable response(s) but no changes were made to the intervention; and (c) *no dissatisfaction*, when results indicated only favorable responses, as an indication that adjustments may not have been needed. Dissatisfaction in rating scales was defined as scores at or below the midpoint (e.g., 3 or lower on a 5‐point scale; Taylor et al., 2019). Table 2 presents a summary of these coding categories. Each of these categories was further coded by timing (i.e., before, during, or after intervention, or unclear) based on when the social validity assessment results were reported.

**TABLE 2:** Definitions and examples of types of intervention adjustments categories.

Types of adjustments	Definition	Example
Adjustment	Social validity assessment results led to the development or modification of an intervention.	Doctoral‐level Board Certified Behavior Analysts assessed the social validity of the training materials using a 5‐point Likert scale and provided feedback, which led to changes in the stimuli before the study (Schnell et al., 2018).
No adjustment to dissatisfaction	Social validity assessment results indicated unfavorable responses, but no changes were made to the intervention.	In an evaluation of a punisher assessment for decreasing automatically reinforced problem behavior, clinicians generally rated the treatment goals as appropriate, the procedures as acceptable, and the outcomes as clinically significant. One clinician noted that the response‐blocking procedure might be difficult to implement with integrity; however, no adjustments to dissatisfaction were noted (Verriden & Roscoe, 2019).
No dissatisfaction	Social validity assessment results only indicated favorable responses.	Adults with intellectual and developmental disabilities who were taught new independent skills via telehealth videoconferencing reported high satisfaction with the goals, procedures, and outcomes, and expressed interest in learning additional skills via telehealth (Pellegrino & DiGennaro Reed, 2020).

Figure 2 illustrates the distribution of articles across social validity assessment timing categories, with stacked bars showing whether social validity assessments led to adjustments, reported no adjustments to dissatisfaction, or reported no dissatisfaction. Adjustments most often occurred when social validity was assessed before intervention. In these cases, researchers used social validity results to guide the development of goals, procedures, or outcomes. For example, Boutain et al. (2020) evaluated a telehealth parent training program for teaching self‐care skills to autistic children. A social validity assessment questionnaire was distributed to parents to rate their satisfaction with their child's ability to perform various skills and the importance of each skill. Based on the results, self‐care skills, such as hand washing and applying lotion, were prioritized for inclusion in the intervention.

The second most common timing for adjustments was during intervention. In these cases, social validity assessment results were used to modify interventions already developed. We found that most studies assessing social validity during intervention evaluated stakeholder preferences for intervention (e.g., using concurrent‐chains arrangements) to identify which procedures to implement during sessions. For example, Halbur et al. (2020) evaluated parents' preferences for different prompting procedures during treatment evaluation using a concurrent‐chains arrangement in which parents selected among written instruction cards corresponding to each prompting strategy before implementing the selected procedure with their child.

The least common timing for adjustments was after intervention or when timing was unclear. In these cases, adjustments to interventions did not occur. It is possible that follow‐up social validity data may have been collected but excluded from manuscripts due to space limitations and that adjustments made after intervention may not have been reported. In contrast, no adjustments to dissatisfaction were most often reported when social validity was assessed after intervention. For example, in a study evaluating the use of internet‐based contingency management to improve adherence with blood glucose testing for teens with Type 1 diabetes, Raiff and Dallery (2010) reported that participants provided less favorable ratings regarding the device and the convenience of the intervention. However, the authors recommended potential improvements based on their participants' feedback in their discussion. Specifically, they suggested making the intervention more convenient by requiring all tests to be submitted at the end of the day instead of throughout the day or by using cell phones to record and submit videos. Reports of no dissatisfaction were also common after intervention.

### Social validity dimensions of adjustments

We examined the dimensions of social validity related to adjustments made in the studies. Supporting Information C provides a summary of the social validity dimensions (i.e., goals, procedures, outcomes, adjusted across different timing assessments). Specifically, we assessed whether researchers adjusted the goals, procedures, or outcomes based on the results of the social validity assessments. For example, if a researcher developed target words for a child based on normative comparison, this would be coded as an adjustment of the goals.

Of the studies that made adjustments following social validity assessments conducted before intervention, adjustments were distributed evenly across all dimensions (i.e., goals, procedures, and outcomes). As an example of an adjustment to the goal dimension, Carlile et al. (2018) conducted social validity assessments before intervention to inform goal selection by observing typically developing peers' help‐seeking responses. These observations were then used to develop task analyses for teaching individuals with autism spectrum disorder to seek help when lost.

Of the studies that made adjustments following social validity assessments conducted during intervention, the adjustments were exclusively focused on procedures, with no changes made to goals or outcomes. For example, Gunning et al. (2020) modified their procedures in the Preschool Life Skills program by creating a “prompt‐sheet” based on parent interviews conducted at the end of each session to identify evocative situations for target skills in natural contexts. No adjustments to dissatisfaction occurred following social validity assessments conducted after intervention or unclear timing of social validity assessments.

### Location of social validity assessment description

We coded the location of the social validity assessment or data description within the manuscript. Supporting Information D provides a summary of whether social validity assessments across intervention timing were described in a specified “Social Validity” section. If the information was presented under a “Social Validity” section, it was coded as such. If not, we noted the specific subheading where the social validity details appeared. For example, if a study assessed participant preferences for intervention using concurrent‐chains procedures under the “Choice Trials” subheading, it would be coded as not being in a dedicated “Social Validity” section.

Social validity assessments conducted after intervention and unclear timing of social validity assessments were most frequently described under a “Social Validity” section. For example, Joslyn and Vollmer (2020) described their social validity assessment conducted after intervention under a “Social Validity” heading in the “Method” section of their study. In contrast, social validity assessments conducted before and during intervention were described under a “Social Validity*”* section in only a small percentage of the studies. Quintero et al. (2020) discussed their social validity assessment conducted before intervention under the “Dependent Variable” heading rather than a “Social Validity” heading. Their social validity assessment involved gathering feedback from experienced coaches on the task analysis to teach correct heading techniques to youth soccer players. Coaches provided input on the wording and accuracy of specific steps in the task analysis.

### Limitations and directions for future reviews

In interpreting these findings, several considerations should be noted. First, our review of the interactive process of social validity was limited to *JABA* articles included in Leif et al. (2024) that reported social validity assessments. Future reviews could expand this scope to include more recent publications and additional journals to more comprehensively evaluate how social validity functions as an interactive process. Although we focused on identifying intervention adjustments as the primary interactive process, perhaps there are other forms of interaction that can be explored (e.g., providing consumers with clear, accessible information about treatment and its rationale) and how such interactions affect social validity data. Future reviews can also evaluate variables that influence whether adjustments are possible and obstacles that may hinder adjustments.

Second, studies may have conducted but not reported social validity assessments before intervention, follow‐up data may have been omitted due to space limitations, and some adjustments after intervention may not have been reported in published manuscripts. Third, other authors have noted additional ways to measure social validity, such as sustained use of procedures (Gast & Ledford, 2014; Kennedy, 2002) and treatment fidelity linked to social acceptability (Allen & Warzak, 2000; Gresham & Lopez, 1996). However, we did not include these measures in our article, as they have not been categorized as social validity assessments in prior reviews. Future researchers could evaluate whether and how these variables function as indicators of social validity.

Another limitation concerns the conceptual considerations regarding choice as a measure of social validity. Some authors have conceptualized choice—particularly choice among interventions or between intervention and no intervention—as an indicator of social validity (e.g., Auten et al., 2024; Vollmer & Pendergrass, 2025). In our review, we considered choice as a measure of social validity specifically when it involved selection among intervention options. Future reviews could examine how choice has been operationalized as a social validity measure. Although such arrangements may provide useful information about acceptability, choice may also be influenced by contextual variables that complicate interpretation. Clarifying when choice reflects procedural acceptability versus other controlling variables may improve conceptual precision in the literature.

Furthermore, we only examined whether social validity assessments appeared in a dedicated “Social Validity” section. Future reviews could investigate where social validity assessments are described across articles and develop clearer guidelines for what qualifies as a social validity assessment. Future research could also examine current social validity applications in service delivery settings and compare them with research.

## CONSIDERATIONS FOR ENHANCING THE INTERACTIVE PROCESS OF SOCIAL VALIDITY

**FIGURE 3:**
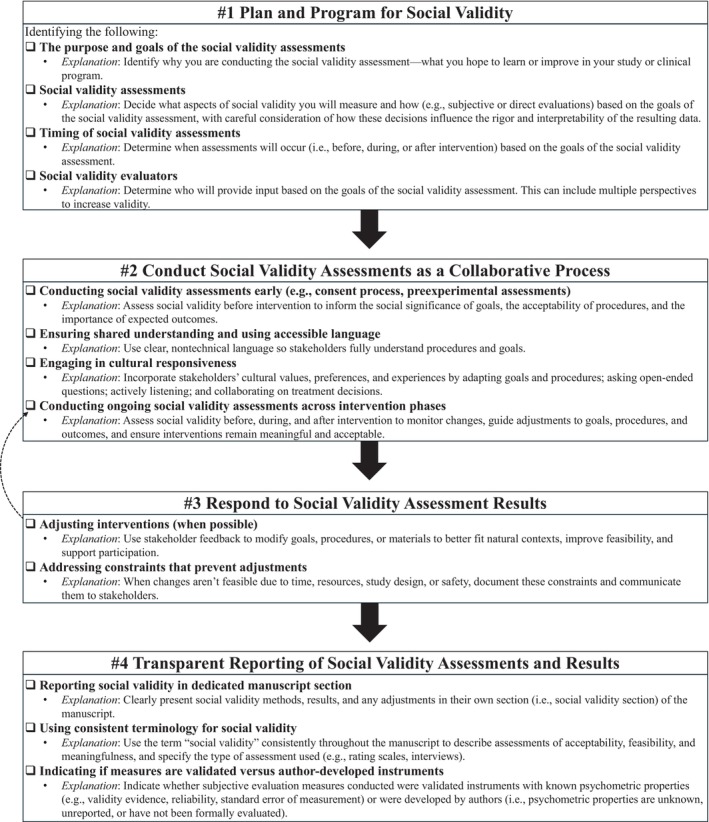
This diagram illustrates a framework for planning, assessing, responding to, and reporting social validity assessments, based on the considerations outlined in this article.

The findings from our review of the interactive process of social validity highlight opportunities to strengthen the interactive process of social validity in research and practice. Although our coding identified patterns in social validity assessment timing and types of adjustments, the results also point to potential variables that may influence the enhancement of social validity as a dynamic, interactive process. We outline considerations and recommendations that researchers and practitioners can apply across intervention phases, followed by general considerations for publication. Figure 3 provides a general overview of these considerations.

### Consideration #1: Plan and program for social validity

Consistent with previous findings (i.e., Kennedy, 1992; Leif et al., 2024), we found that most studies conducted social validity assessments after intervention. Instead of measuring social validity as an afterthought, programming for social validity ensures a more intentional approach to measuring and using these data to inform changes to interventions. To program for social validity, researchers can plan for social validity by identifying (a) the purpose and goals of the social validity assessments for the study, (b) what to measure and how to measure it, including considerations related to the rigor and interpretability of the assessment methods and resulting data, (c) when to measure it, and (d) who provides input. Furthermore, we did not provide specific recommendations for these variables, as they should ultimately be guided by the study's purpose and the decisions researchers seek to inform.

#### Identify the purpose and goals of the social validity assessments

If researchers and practitioners determine that conducting a social validity assessment is beneficial, they may begin by clarifying its purpose and goals. These assessments may serve different functions depending on the context and the types of decisions they are intended to inform (Finn & Sladeczek, 2001). For example, a study evaluating the effectiveness of two interventions may define its social validity purpose as assessing stakeholders' preferences between those interventions. Based on this goal, researchers may use a treatment preference evaluation (e.g., concurrent‐chains procedure) as a direct evaluation of social validity.

Other purposes of social validity assessments may include evaluating whether intervention goals are meaningful to stakeholders, whether procedures are acceptable and feasible to implement, or whether intervention outcomes are perceived as beneficial. Because the purposes differ, they require different types of social validity assessments. For example, in one of the studies reviewed in this article, Garcia‐Albea et al. (2014) reported that the purpose of their social validity assessment was to determine whether participants were perceived as engaging in more appropriate language following the intervention. They designed their social validity assessment to evaluate this outcome by presenting video clips from baseline and maintenance sessions to college students who served as raters. Clearly defining the purpose of the social validity assessment can help guide subsequent decisions regarding what aspects of social validity to measure, when assessments should occur, and who should provide input.

#### Identifying social validity assessments

Planning for social validity requires selecting assessments that match the intended construct and provide actionable information. Such selection can also reduce the likelihood of obtaining unreliable or uninformative results (Carr et al., 1999) and support meaningful intervention adjustments. Researchers may use subjective evaluations (e.g., rating scales), direct evaluations (e.g., treatment preference evaluation), or a combination of both. Importantly, the selection of a specific social validity assessment should be guided by its intended function (e.g., evaluating acceptability, feasibility, preference, satisfaction), with assessment type and implementation aligned accordingly.

##### Subjective evaluations

Because social validity reflects stakeholder judgements and is inherently subjective (Wolf, 1978), its assessment often relies on subjective evaluation methods. Subjective evaluations are particularly appropriate when the goal of the social validity assessment is to identify potential barriers to implementation, inform early adjustments before intervention begins, or assess stakeholder perceptions of the intervention (e.g., acceptability, feasibility, importance). However, because these methods rely on stakeholder reports, they are susceptible to response bias (e.g., if rating scales are not completed anonymously, stakeholders may provide more favorable ratings to avoid negatively evaluating interventionists).

Subjective evaluations may be unstructured or structured, with structured measures further categorized as validated or unvalidated. Unstructured subjective evaluations, such as anecdotal reports and informal interviews, may provide rich contextual information regarding stakeholder experiences, preferences, concerns, and implementation constraints that may not be captured through standardized questionnaires (Gresham & Lopez, 1996). For example, a clinician may engage in informal conversations with a caregiver about the feasibility of implementing intervention procedures during daily routines or ask open‐ended questions regarding concerns about intervention goals. Although unstructured approaches can capture information that is not readily accessible using more structured methods (e.g., rating scales), these approaches are subject to interviewer and respondent biases and may not be reproducible.

Structured subjective evaluations (e.g., rating scales, structured interviews) provide a more systematic approach to assessing social validity and may be either validated or unvalidated (i.e., developed by the authors). Author‐developed measures are those for which psychometric properties are unknown, unreported, or have not been formally evaluated. They may be useful when researchers seek to address context‐specific concerns that may not be captured by existing instruments (e.g., Treatment Acceptability Rating Form; Reimers & Wacker, 1988). For example, a study evaluating habit reversal to decrease filled pauses in public speaking used an author‐developed 5‐point Likert rating scale to assess participants' perceptions of their public speaking ability, comfort, and confidence (Mancuso & Miltenberger, 2016). Although author‐developed measures allow for flexibility and customization, they assess constructs that are not clearly defined or that lack evidence of reliability and validity, which limits confidence in the interpretation of social validity results (i.e., whether the data accurately reflect the relevant dimensions of social validity).

The field of applied behavior analysis emphasizes rigorous data, typically achieved through objective and precise measurement of behavior; this emphasis also applies to social validity. Accordingly, subjective evaluations of social validity—which assess inherently subjective constructs—are expected to meet similar standards of rigor when used to inform research and practice. Ensuring that subjective evaluations accurately capture the intended construct requires consideration of psychometric principles. Psychometrics is the science concerned with evaluating the properties of psychological measures (e.g., rating scales; Furr, 2022) and helps determine whether they measure what they are intended to assess. For example, respondents for a social validity assessment questionnaire may provide favorable ratings because they liked the interventionist or appreciated the time and effort provided rather than because the intervention procedures or outcomes were perceived as acceptable. Developing and validating such measures is critical in health, social, and behavioral research and involves specific, systematic techniques (Boateng et al., 2018). However, behavior analysts may receive limited formal training in psychometric science, which may affect the selection and interpretation of social validity assessments. As a result, careful consideration of assessment tools is warranted, particularly when subjective evaluations are used to support conclusions about social validity.

One way to support rigor in subjective evaluations is through the use of validated measures. Validated measures have established evidence regarding reliability and validity, increasing confidence that the assessment is measuring the relevant dimensions of social validity. Examples of validated measures that have been used to assess behavioral intervention acceptability and related constructs include the Treatment Evaluation Inventory (Kazdin, 1980), Treatment Evaluation Inventory–Short Form (Kelley et al., 1989), Intervention Rating Profile–15 (Martens et al., 1985), Behavior Intervention Rating Scale (Elliott & Treuting, 1991), and Treatment Acceptability Rating Form (Reimers & Wacker, 1988). Moreover, measurement error inherent to rating scales can be quantified and considered to determine whether differences in scores (across time or conditions) reflect actual differences in acceptability rather than measurement variability. For example, changes in caregiver ratings from before to after intervention may be interpreted as meaningful only when they exceed the amount of variation expected due to measurement error. More accurate interpretation of social validity findings strengthens the conclusions drawn from these data and increases the relevance of findings for research and practice within and beyond applied behavior analysis.

Furthermore, subjective evaluations can also be strengthened by presenting structured and observable materials to social validity evaluators, such as videos of intervention sessions. For example, researchers or clinicians may show caregivers a short video of a child engaging in a play activity after intervention and ask targeted questions about the acceptability of procedures, the child's engagement, or the perceived meaningfulness of the outcomes. Providing this context may facilitate more informed social validity evaluations. However, these social validity assessments remain subjective because they rely on evaluators' interpretations rather than direct behavioral measures. Thus, subjective and direct evaluations can also be conceptualized along a continuum, with supplemental structured observations of the intervention (e.g., videos) providing context that makes subjective input more informed. Although structured and observable materials can enhance subjective evaluations, some aspects of social validity may be best captured through direct evaluations.

##### Direct evaluations

Direct evaluations may be particularly useful when social validity is assessed through observable behavior or outcomes that indicate judgements of acceptability, preference, or alignment with socially desirable standards. When planning direct evaluations of social validity, researchers and clinicians can begin by considering the specific goal of the assessment, as different methods capture different aspects of social validity. However, it is also important to consider whether a normative goal is socially valid for a given participant or client. This issue has been a point of contention, particularly in the treatment of individuals diagnosed with autism spectrum disorder (Ne′eman, 2010). Typically, normative comparisons for individuals with autism involve benchmarking performance against age‐matched peers; however, this comparison may not always reflect meaningful or appropriate goals or outcomes for all individuals. In some cases, comparing participants with development‐matched peers may be more appropriate than comparing them with age‐matched peers. For example, previous studies on teaching play skills have found that children with autism reached criterion and demonstrated generalization more consistently with developmentally appropriate play targets than with age‐appropriate targets (Agana et al., 2024; Lifter et al., 1993; Pane et al., 2022). Thus, a development‐matched approach may, in some cases, represent a more socially valid alternative to age‐based normative comparisons.

Alternatively, if the goal of social validity assessment is to evaluate participants' preferences for specific interventions, concurrent‐chains assessments may be appropriate. For example, a researcher might arrange a concurrent‐chains procedure in which a child chooses between two play interventions, one with video modeling and the other with in vivo modeling. However, concurrent‐chains arrangements may also be susceptible to bias. For example, selection of a condition (e.g., intervention) may be influenced by variables other than preference for that condition (e.g., Owen et al., 2021; Rodriguez et al., 2024). As a result, it is important to consider whether observed responding reflects true preference for a treatment or is influenced by contextual variables.

In addition to the methods described above, there are other direct evaluation approaches that may be conceptualized as indicators of social validity but have received limited attention in the literature concerning social validity assessments. Two examples are assessing (a) assent and (b) sustained use of an intervention as social validity indicators. Although these methods were not included in our review, they may provide valuable information about the acceptability and meaningfulness of interventions from the perspective of the direct recipient.

When the goal of social validity assessment is to determine whether an individual is willing to participate in an intervention, assessing assent may be informative. The Behavior Analyst Certification Board (2020, p. 7) defines assent as the “vocal or nonvocal verbal behavior that can be taken to indicate willingness to participate in research or behavioral services by individuals who cannot provide informed consent (e.g., because of age or intellectual impairments).” Assent may also be conceptualized as a form of choice (i.e., whether to participate in an activity or not), which allows researchers or clinicians to evaluate willingness to engage (Morris et al., 2021). However, the idea of “free choice” can be misleading from a behavior‐analytic perspective, as all behavior occurs under environmental influence (Bannerman et al., 1990). We refer readers to Morris et al. (2024) for suggestions on framing choices as fair choices, arranged to minimize undue influence while recognizing contextual control. Assent can also be measured through observable behavior that indicates willingness to engage. That is, assessing assent as an indicator of social validity may include tacting that an establishing operation is present to continue an activity by observing an indicating response (Frampton et al., 2024) such as the participant initiating, reaching, approaching, or making eye contact toward or with the research materials or experimenter. In contrast, dissent or loss of assent may include the opposite of an indicating response such as avoidance, withdrawal, increased distress, or nonparticipation.

Relatedly, choice at the level of intervention selection, particularly choice among interventions or between intervention and no intervention, has been conceptualized as a measure of social validity (e.g., Auten et al., 2024; Vollmer & Pendergrass, 2025) and was therefore included in the present review. But given the limitations associated with choice, sustained use of procedures (i.e., continued posttreatment implementation of procedures; Gast & Ledford, 2014), may be an alternative option to determine acceptability of an intervention. Continued implementation by direct stakeholders after intervention is withdrawn suggests that procedures are perceived as acceptable, feasible, and worthwhile. For example, if caregivers or teachers continue to implement an intervention after external support is withdrawn, this pattern of use may reflect high social validity even in the absence of concurrent‐chains arrangement to evaluate preference of an intervention. Although some authors (e.g., Common & Lane, 2017; Gast & Ledford, 2014) have recommended measuring social validity through sustained use of procedures, it remains unknown how often this approach is used in research and practice. It should also be noted that discontinuing use of procedures following an intervention may occur for a variety of reasons, including not only low social validity but also fluency with the intervention (e.g., teachers are briefly taught an intervention but may not have been taught until demonstration of proficiency) or issues with generalization (e.g., trained insufficient exemplars; Allen & Warzak, 2000).

##### Social validity dimension

In addition to selecting social validity assessment formats and measurement strategies, researchers and clinicians can also consider the specific dimension of social validity being evaluated (i.e., goals, procedures, or outcomes). Identifying the relevant dimension can guide the selection of social validity assessments that provide meaningful information for a study's purpose. For example, evaluating the acceptability of procedures may be particularly important when caregivers or teachers are responsible for implementation. In contrast, assessing the appropriateness of goals may be more relevant when comparing different approaches to target selection. Selecting complementary social validity assessment methods across dimensions may also provide a more detailed understanding of stakeholder perspectives.

#### Identifying timing for social validity assessments

Once researchers determine the types of social validity assessments to use and the dimensions they will address, the next consideration is when to conduct them. The timing of social validity assessments can influence the interactive process of social validity. Although incorporating social validity assessments across all stages of an intervention (i.e., before, during, and after intervention) is ideal, practical limitations may prevent this. In such cases, the timing of social validity assessments can be guided by the study's purpose because doing so increases the likelihood that the collected information will inform meaningful intervention decisions. For example, social validity assessments conducted before and during intervention may be especially useful for gathering subjective evaluations (e.g., anecdotal reports) when collaboration in intervention development (e.g., goal selection) is a priority. For example, in one study teaching kindergarten students how to respond during lockdown drills, researchers gathered social validity data before intervention from the local police department and school administration, who preferred that all students receive intervention (rather than no intervention) regardless of individual performance. These anecdotal reports guided the intervention procedures and supported the study's purpose of teaching kindergartners effective lockdown responses (Dickson & Vargo, 2017).

In contrast, social validity assessments conducted after intervention may facilitate less collaboration in the interactive process; we found no reported adjustments to dissatisfaction made at this stage (further described in the section on “Addressing constraints to adjustments”). Nevertheless, social validity assessments conducted after intervention can still provide meaningful information that may align with a study's purpose. For example, direct evaluations of outcomes (e.g., treatment preference evaluations when comparing two or more interventions) may be particularly valuable for understanding which procedures or results are most acceptable or preferred. Subjective evaluations conducted after intervention can also capture consumer perspectives on the intervention's overall efficacy and may be strengthened by showing videos or other materials that help contextualize the outcomes being evaluated.

Furthermore, other fields (e.g., psychology, medicine, implementation science) also emphasize assessing stakeholder (e.g., patient) perspectives across different stages of an intervention. These disciplines examine constructs related to social validity (e.g., acceptability, feasibility, preference, satisfaction) and consider the timing of assessments. For example, acceptability has been conceptualized from two temporal perspectives—prospective (forward‐looking) and retrospective (backward‐looking)—and can be assessed at similar stages (i.e., before, during, or after intervention; Sekhon et al., 2017). The timing of assessments may also vary depending on their purpose. Specifically, acceptability may be evaluated either before or after intervention, whereas satisfaction is typically assessed following exposure. Similar to our emphasis on specifying the purpose of social validity assessments, Sekhon et al. (2017) highlighted the importance of clearly defining the purpose of acceptability assessments at different points and aligning that purpose with the temporal perspective being adopted.

#### Identifying social validity evaluators

Another important consideration in planning for social validity assessments is determining who serves as evaluators (e.g., participants, caregivers, teachers) because their perspectives can influence the relevance and usefulness of the social validity assessment results. Consumers, consultants, and broader segments of society may hold differing perspectives regarding treatment goals (Carter & Wheeler, 2019). For example, Schwartz and Baer (1991) identified four classes of consumers: direct, indirect, members of the immediate community, and members of the extended community. Their framework underscores that many individuals beyond direct recipients can act as passive consumers of treatment programs. Furthermore, although opinions from these groups may not always align, they provide perspectives on how goals are perceived and valued across audiences.

Gathering input from multiple evaluator types may help guide treatment goals in ways that are meaningful to more than one social category, particularly for stakeholders responsible for implementing or maintaining them after research. Other researchers also highlighted expert judges, or professionals with knowledge relevant to the treatment context, as an additional valuable source of perspective (e.g., Kazdin, 1977). Importantly, Schwartz and Baer (1991) emphasized that if social validity assessments provide information that promotes program viability, then input from consumers most directly tied to program success can be given the greatest weight.

Furthermore, social validity ratings may differ across evaluator types, particularly when interventions require sustained effort or coordination among multiple stakeholders. For example, in school‐based interventions designed to support students' engagement in instructional routines or reductions in disruptive behavior, caregivers may rate intervention goals as highly acceptable because they align with long‐term academic or social outcomes, whereas special educators may view the procedures as less acceptable due to time constraints, competing classroom demands, or staffing limitations. In such cases, gathering input from multiple evaluators can help researchers and practitioners identify differing perspectives and whether modifications to procedures, role assignments, or delivery contexts are needed to support feasibility.

### Consideration #2: Conduct social validity assessments as a collaborative process

Previous reviews (e.g., Leif et al., 2024) have recommended ongoing social validity assessments at various stages of the intervention process. Extending these recommendations, we conceptualize social validity assessment as a collaborative process involving researchers, practitioners, and stakeholders. Rather than occurring only after intervention, social validity assessments can be integrated throughout the intervention process to inform planning, implementation, and evaluation. Conducting social validity assessments collaboratively may allow researchers and practitioners to gather stakeholder perspectives early, ensure shared understanding of intervention goals and procedures, incorporate cultural and contextual considerations, and monitor perceptions across intervention phases. In our review, we found that social validity assessments conducted earlier in the intervention process were more frequently used to guide intervention development than those conducted during or after intervention phases. The sections below describe several practices that may support a collaborative approach to social validity assessment including (a) conducting assessments early in the process, (b) ensuring shared understanding through accessible language, (c) engaging in culturally responsive practices, and (d) conducting social validity assessments across intervention phases to monitor stakeholder perspectives over time.

#### Conducting social validity assessments early

One approach to early social validity assessments, as recommended by Leif et al. (2024), is during the informed consent process, which can provide an opportunity to gather social validity data prior to implementation of intervention. Another method is through preexperimental assessments, which we found were often described under headings such as “Preassessments” or “Selection of Target Skills.” These contexts represent naturally occurring opportunities to obtain stakeholder input before intervention without requiring substantial additional social validity assessment procedures. Although we did not examine social validity in practice or clinical work, conducting early social validity assessments in the research process mirrors the intake process in applied settings. Perhaps a proactive approach could also lead to improved social validity outcomes after intervention.

If researchers explicitly report any social validity assessments during the informed consent process or preexperimental assessments—whether structured or unstructured—this may provide a more complete understanding of how social validity assessments inform intervention development. These assessments may have been underreported in the past, perhaps because they were seen as part of routine procedures (e.g., obtaining consent from participants or caregivers) rather than an avenue to conduct social validity assessments. Recognizing and reporting these practices more consistently can provide a fuller picture of how social validity influences program development, particularly before intervention.

#### Ensuring shared understanding and using accessible language

Early social validity assessments, particularly before intervention, not only inform intervention design but also create opportunities for shared understanding and reciprocal learning among researchers, practitioners, and consumers. Hawkins (1991) noted that one purpose of social validity measures is to identify when additional education for consumers may be necessary. However, we highlight that rather than serving solely to educate consumers, social validity assessments can support collaborative communication by identifying areas for which clarification, dialogue, or additional information may be needed to meaningfully evaluate intervention goals, procedures, and outcomes.

Ensuring shared understanding can be considered a core component of an interactive social validity process, as a clear understanding of an intervention provides a foundation for evaluating its acceptability and may increase opportunities for meaningful feedback from consumers and stakeholders. Reimers et al. (1992) emphasized this in a flowchart‐based model showing that comprehension guides decisions across implementation outcomes. Limited shared understanding may contribute to reduced engagement or inconsistent implementation, underscoring the importance of establishing clear communication before acceptability can be fairly evaluated. Without this shared understanding, early social validity assessments may indicate low acceptability that reflects misunderstanding or incomplete information rather than true rejection of the intervention. Distinguishing between these possibilities is critical for interpreting early social validity data. For example, caregivers may request behavior changes that conflict with established evidence. Within a collaborative framework, these situations can be approached as opportunities for dialogue in which practitioners share relevant empirical support while also attending to caregivers' goals, values, and concerns. Such discussions may involve jointly reviewing the evidence supporting or contradicting specific targets (Rohrer et al., 2021; Rohrer & Weiss, 2023) while remaining responsive to the contextual factors influencing acceptability. Although this section emphasizes shared understanding as a prerequisite for evaluating acceptability, broader considerations related to learning from consumers' lived experiences, cultural contexts, and values are discussed in greater detail in the section on “Engaging in cultural responsiveness.”

Furthermore, providing information in accessible, lay‐language terms supports both shared understanding and acceptability. Scientific fields, including behavior analysis, rely on technical terminology for precision among professionals (Normand, 2019); however, jargon can create confusion or resistance for lay audiences (Friman, 2021). For example, caregivers are unlikely to adopt terms like “mand” or “motivating operation” outside professional contexts (Friman, 2021). Defining treatment goals in both clinical and lay terms increases social validity by making them meaningful to those closely involved (Carter & Wheeler, 2019). For example, a goal stated as “increase mands across two settings by 30%” may be clear to clinicians, but caregivers may better understand “This intervention will teach your child to independently ask for items and activities they want.” Consumers or clients often report greater comfort and confidence when procedures are clearly communicated (Vahdat et al., 2014). Furthermore, accessible language may help ensure that caregivers recognize when treatment goals are achieved as well as improve the accuracy of subjective social validity assessments.

#### Engaging in cultural responsiveness

When planning and programming for social validity, researchers and practitioners can intentionally incorporate strategies that make interventions more meaningful, acceptable, and relevant to consumers. One strategy that may support this process is engaging in culturally responsive practices during the assessment of social validity. Cultural responsiveness involves continuously identifying and discriminating cultural variables in the environment (e.g., language, ethnicity) that influence behavior, understanding how these stimuli influence learning histories, and adapting assessment and intervention practices accordingly (Jimenez‐Gomez, 2024; Jimenez‐Gomez & Beaulieu, 2022). Cultural responsiveness can be applied across the three domains of social validity by selecting culturally anchored goals, using culturally appropriate procedures, and evaluating outcomes based on alignment with everyday values and practices. These practices can also facilitate attention to environmental contingencies (e.g., cultural variables that influence behavior) and reduce making generalizations within the assessment and treatment process (Vogel et al., 2025). We refer readers to Jimenez‐Gomez and Beaulieu (2022) for a comprehensive overview of this topic.

Jimenez‐Gomez and Beaulieu (2022) highlighted several culturally responsive strategies that support behavior analysts with conducting social validity assessments collaboratively with stakeholders including (a) adapting treatments based on cultural variables, (b) asking open‐ended questions and actively listening to caregiver concerns, (c) collaborating with caregivers on treatment goals and treatment selection, and (d) offering choices of treatment components. These strategies can be implemented in social validity assessment practice in several ways. First, researchers and practitioners could adapt social validity assessments to consider cultural variables. For example, during social validity assessments, behavior analysts might ask caregivers whether proposed treatment goals align with family priorities or routines or inquire about cultural practices, beliefs, or values that could influence behavior and the effectiveness of care. Specific questions might include the following: “Are these goals consistent with your family's daily routines?” or “Are there any family practices or expectations that we should consider when selecting targets or intervention strategies?” Such questions could help with adapting treatments based on cultural variables. These practices are particularly important given findings that culturally informed collaboration is often limited; for example, Beaulieu et al. (2018) reported that only 39% of practitioners consistently inquired whether treatment goals aligned with family priorities, whereas 30% reported asking caregivers this question infrequently or not at all.

Second, asking open‐ended social validity questions and actively listening to caregiver concerns allows caregivers to identify priorities and share information about routines or values that may otherwise be overlooked. This may involve using open‐ended rather than closed questions (e.g., “yes” or “no”), restating or summarizing caregiver responses to check understanding, and avoiding leading or multipart questions that could constrain caregiver input. For example, in a sleep intervention directed at establishing a bedtime routine, a behavior analyst might ask before intervention, “How would you know if a new bedtime routine is helpful for your child?” or “Are there any family practices around bedtime that we should consider?” After the caregiver responds, the behavior analyst might summarize the response (e.g., “I want to make sure I understood correctly—could you tell me whether I captured your concerns accurately?”) and invite clarification if needed. This process allows the behavior analyst to better understand caregiver priorities and use that input to guide collaborative intervention planning. Third, collaborating with caregivers on treatment goals and intervention selection can involve offering multiple options for procedures, materials, or contexts and jointly deciding which are most feasible and relevant. For example, when creating the bedtime routine for intervention, caregivers might be offered options for the sequence of activities, types of materials used (e.g., books, music), or timing of routines and asked to select what best fits their family's schedule and preferences. We discussed the limitations of choice as a measure of social validity earlier; however, offering options to support culturally responsive practices may increase families' perception of the treatment's social validity when evaluated after intervention.

Cultural responsiveness is distinct from social validity itself but can serve as a mechanism for enhancing it. For example, collaborating with caregivers—such as by asking open‐ended questions related to how goals fit in their everyday lives and actively listening to their concerns during goal selection (an example of engaging in cultural responsiveness)—can increase the relevance of intervention goals, whereas social validity reflects the extent to which stakeholders perceive those goals and procedures as acceptable, feasible, and meaningful. Using culturally responsive practices in social validity assessment and planning allows researchers and practitioners to design interventions that align with stakeholder cultural values and are more likely to be considered meaningful and socially valid.

#### Conducting ongoing social validity assessments across intervention phases

Opportunities to respond to social validity assessments increase when these assessments are conducted throughout the study. To extend our earlier recommendation to assess social validity early in the research process, we propose conducting social validity assessments continuously across all phases of intervention (before, during, and after intervention). We found that most adjustments occurred before and during intervention phases. Adjustments made before intervention primarily involved using social validity assessment results to inform intervention development. This may be because conducting social validity assessments before intervention is more conducive to intervention development. In contrast, adjustments made during intervention involved changing an already‐developed intervention based on social validity results. Additionally, we found that adjustments occurred across all dimensions (i.e., goals, procedures, outcomes) before intervention, that only procedures were adjusted during intervention, and that no adjustments to dissatisfaction occurred after intervention. Our findings suggest that social validity assessments conducted before and during intervention may facilitate an interactive process that leads to adjustments to the intervention and additional dimensions being addressed. According to Ndanganeni et al. (2025), measuring social validity only after intervention maintains the existing power dynamic between the researcher or interventionist and the consumer, which keeps the decision‐making authority primarily with the professionals.

Several practical constraints can make continuous social validity assessment challenging. Limited personnel, time, or access to resources (e.g., video equipment, digital survey platforms) may restrict frequent assessments, particularly in labor‐intensive interventions or multisite studies. For example, caregivers, participants, or behavior‐change agents may have limited availability, and repeated surveys or interviews can lead to fatigue (Jeong et al., 2023) and less thoughtful responses. Dynamic contexts, including staffing changes or family routines, can also influence the acceptability and feasibility of interventions over time. Considering these constraints allows researchers and practitioners to plan social validity assessments in ways that align with the timing and frequency with the specific intended purpose of the social validity assessments (e.g., evaluating acceptability of the procedures versus meaningfulness of outcomes). For example, if researchers have identified that evaluating the social validity of the study's procedures is the priority, brief social validity assessments before can establish baseline acceptability, whereas assessments during intervention can monitor how acceptability evolves once the intervention is underway. By planning each social validity assessment around its specific purpose, researchers and practitioners can tailor when, how often, and how it is conducted to gather actionable information rather than requiring intensive social validity measures or overly burdensome data collection at every stage.

### Consideration #3: Respond to social validity assessment results

Finney (1991) proposed that social validity in behavioral research is an interactive process in which researchers implement interventions, consumers provide feedback, and researchers adjust interventions (but not always) accordingly. However, there is limited guidance on implementing this interactive process. Although there is no empirical basis for specific recommendations on when to adjust interventions, we discuss how the results of social validity assessments can be used to inform actions to enhance social validity.

#### Adjusting interventions (When possible)

When the results of a social validity assessment yield unfavorable responses (i.e., dissatisfaction) to the intervention or specific components, researchers or practitioners can first determine whether adjustments are possible. As noted earlier, dissatisfaction may sometimes reflect limited shared understanding of the procedures by the researchers or clinicians and the stakeholders, in which case brief clarification may resolve concerns. However, if concerns persist, adjustments to the intervention may be considered.

Adjustments to interventions may be possible if a study is evaluating the effectiveness of an intervention rather than its efficacy. Singal et al. (2014) described that efficacy research is conducted in controlled settings with homogeneous groups, using well‐defined treatments delivered with high fidelity by researchers for a short period. Effectiveness research, in contrast, occurs in natural settings (e.g., schools, homes) with more heterogeneous populations, using multicomponent interventions implemented by caregivers with varying fidelity typically over longer periods (Singal et al., 2014). Consequently, researchers studying effectiveness may place greater emphasis on collaborating with stakeholders, such as caregivers, to identify factors that support successful implementation. For example, Mendres‐Smith et al. (2020) evaluated a parent‐led tummy time intervention in which mothers interacted chest‐to‐chest with their infants using a toy. Beginning at session nine, the experimenter terminated sessions after 1 min of negative vocalizations because a child's mother reported high distress when hearing him cry and occasionally left the room. To address this, the experimenter collaborated with the mother to identify a low‐effort activity she could engage in during sessions, such as checking emails, which was based on previous research suggesting that distracting activities can reduce the aversive nature of infants' cries (e.g., Glodowski & Thompson, 2017). After implementing this adjustment, the mother reported that she tolerated her child's cries better. This example depicts how interventions can be adjusted to support caregiver implementation and enhance social validity that is guided by previous research.

In research, unfavorable social validity results could be addressed by increasing the ecological validity of an intervention. Ecological validity refers to the extent to which research conditions and procedures reflect how behavior and interventions occur in real‐world contexts (Schmuckler, 2001). Although ecological validity does not equal social validity, designing interventions with high ecological validity can positively influence consumers' perceptions of their acceptability and feasibility (i.e., social validity). For example, designing interventions in collaboration with family members, situated within familiar contexts and using naturally occurring contingencies and materials (e.g., household items), may increase the acceptability, feasibility, and perceived authenticity of intervention procedures. Fahmie et al. (2023) suggested that involving stakeholders (e.g., caregivers, participants) in decisions about study design can enhance ecological validity by ensuring that interventions more closely resemble preferred interactions under naturally occurring conditions. Such adjustments may simultaneously improve ecological validity and increase the likelihood that intervention procedures are viewed as meaningful, feasible, and acceptable by consumers.

Another example of when researchers may adjust interventions is through collaboration with experts in a specific treatment or tool to increase the feasibility and implementation of procedures. For example, Mitteer et al. (2018) consulted Board Certified Behavior Analysts with experience graphing single‐case design data in Prism to establish mastery criteria and session durations for teaching behavior technicians to create publication‐quality graphs. Experts completed target graphs using preentered data, and their performance informed both the study's mastery criteria and maximum session duration. Furthermore, in multidisciplinary research, input from experts in other fields can be valuable. Such collaboration could help ensure procedures are appropriate, feasible, and aligned with best practices.

These examples illustrate several ways researchers and practitioners may respond to unfavorable social validity results including collaborating with stakeholders to adjust procedures, increasing ecological validity, and consulting experts to enhance feasibility and implementation. These strategies are not intended to represent an exhaustive list but rather to highlight potential avenues for responding to social validity feedback and to encourage further discussion and refinement of these practices. After adjustments are made, researchers and practitioners may consider returning to the process of conducting social validity assessments to evaluate whether the modifications result in improved perceptions of social validity.

#### Addressing constraints that prevent adjustments

Even when social validity assessments reveal dissatisfaction or concerns, researchers and practitioners may face challenges hindering intervention adjustments. We found no adjustments to dissatisfaction after intervention, likely due to difficulties fostering an interactive process at this stage. For example, some studies used anonymous questionnaires after the intervention concluded (e.g., Sump et al., 2018), limiting adjustments because participant dissatisfaction remained unknown. Addressing concerns after the intervention can also be resource intensive. For example, graduate students may face time constraints with respect to responding to after‐intervention results once the study is completed as part of a student's thesis or dissertation. It is also possible that adjustments after intervention are not reported due to space limitations in the manuscript or because researchers consider such adjustments to extend beyond the primary purpose of the study. Perhaps researchers could consider including additional follow‐up social validity data to further inform intervention adjustments and support the interactive process beyond the intervention period.

When adjustments are not feasible, researchers can still respond by documenting constraints and using feedback to recommend future research that may address the dissatisfaction (e.g., Raiff & Dallery, 2010). Importantly, even for well‐established or highly efficacious treatments, researchers and practitioners can consider modifying ancillary aspects of the intervention (e.g., frequency, timing, setting) based on social validity results. Such adjustments can enhance the perceived feasibility, acceptability, and stakeholder satisfaction without altering the core components of the intervention. Maintaining a record of these decisions, noting that they were informed by social validity data, increases transparency and provides guidance for future research on balancing effectiveness with social validity.

### Consideration #4: Transparent reporting of social validity assessments and results

As we advance our field in the assessment and enhancement of social validity, it is important to carefully consider where (i.e., location in the manuscript) and how (e.g., terminology used) social validity assessments are reported. Clear and consistent reporting could help ensure that these data are accurately identified, interpreted, and incorporated into the literature. Transparency can be enhanced by (a) reporting social validity assessments in clearly designated sections, specifying whether they occur before, during, or after intervention; (b) consistently using the term “social validity” when presenting qualitative or quantitative data on goal significance, procedural appropriateness, or outcome importance; and (c) explicitly indicating whether social validity measures used were validated instruments with known psychometric properties (e.g., validity evidence, reliability, standard error of measurement) or author‐developed measures.

#### Reporting social validity in dedicated manuscript sections

Clearly reporting social validity assessments and adjustments within dedicated sections can better ensure that their role in evaluating social validation is well understood. We found that social validity assessments conducted after intervention were predominantly reported under the subheading of “Social Validity,” whereas this was not consistently the case for social validity assessments conducted before or during intervention. This suggests that social validity assessments conducted before and during intervention may have been underreported in previous reviews.

Specifying when social validity assessment and adjustments are conducted (i.e., before, during, or after intervention) provides readers with important information and will facilitate replication. It can also be helpful to link each social validity assessment to any resulting intervention changes. This practice can standardize social validity reporting as well as making it easier for future researchers to identify and interpret these social validity assessments accurately. It may also potentially influence whether and how these assessments are prioritized in our field. Furthermore, Finney (1991) recommended that behavioral journals provide guidelines on when to report social validity data during the publication process. Journal guidelines could improve clarity concerning what, when, and how to include social validity measures. Additionally, underreporting may also result from ambiguity in recognizing which procedures qualify as social validity assessments.

In addition to reporting social validity in dedicated sections, attention to detailed scenarios can enhance the interpretability and utility of social validity data. For example, different types of social validity evaluators (e.g., caregivers, teachers, behavior analysts) could be reported separately rather than aggregated, as differences in perception can provide important insights across evaluator types. Researchers could also be transparent about contextual or logistical constraints that may have influenced social validity measurement or reporting, such as manuscript space limitations or anonymization requirements for evaluators.

#### Using consistent terminology for social validity

Inconsistent terminology also contributes to difficulties in identifying social validity assessments. For example, Huntington et al. (2023) found that although “social validity” was the most commonly used term, others (e.g., acceptability) were also used to describe the same concept. This may have contributed to some of the differences obtained in our review and those of Leif et al. (2024). To reduce confusion, consistently using the term “social validity” while also specifying the type of assessment used (e.g., rating scales, interviews) can improve the identification and interpretation of qualitative or quantitative data on the significance of goals, appropriateness of procedures, or importance of outcomes (Wolf, 1978). In addition, when relevant, researchers may specify the particular construct being targeted within social validity (e.g., treatment acceptability, consumer satisfaction, perceived effectiveness), particularly when these terms are used in other fields (Sekhon et al., 2017) or aligned with established measures. However, these more specific constructs may be best framed as aspects of social validity to maintain conceptual consistency across behavior‐analytic literature. Additionally, researchers could improve transparency by clearly describing the procedures used to assess social validity, including who served as evaluators, when the assessment occurred, how it was administered, and what aspects of social validity were measured. For example, authors might report that “social validity assessment was conducted after intervention to evaluate caregiver perceptions of treatment acceptability of intervention procedures and satisfaction with outcomes by having parents complete a rating scale.”

#### Indicating whether measures are validated versus developed by the author(s)

To further enhance transparency and interpretability of social validity data, it is important that researchers explicitly indicate whether subjective evaluation measures (e.g., rating scales, questionnaires, structured interviews) are validated instruments or author‐developed measures. This distinction is important because it provides readers with information regarding the degree of psychometric support for the measure (Furr, 2022), which increases confidence that the findings provide a measure of social validity rather than a different construct (e.g., general appreciation for the intervention or the researcher's efforts) and that differences in scores across conditions or time are due to actual changes in social validity rather than fluctuations in scores due to measurement error. With respect to research, the use of validated measures permits comparisons of findings across studies, which could facilitate replication studies, literature reviews, and meta‐analyses.

When validated instruments are used, researchers citing the original source, describing the purpose for which the instrument was developed, and reporting available psychometric evidence relevant to its use (e.g., validity, reliability or internal consistency, standard error of measurement) can further support interpretation and comparison findings. Although not included in our review, Braren et al. (2026) provided this type of information in their report of a social validity assessment. Specifically, they reported using a modified version of the Intervention Rating Profile–15 (Martens et al., 1985), an empirically validated measure of teacher acceptability of behavioral interventions. They also noted that the full Intervention Rating Profile–15 demonstrated high reliability and good concurrent validity in the original study as well as acknowledging that the psychometric properties of their modified 11‐item version were unknown and that the findings should therefore be interpreted with caution. If the standard error of measurement is known, reporting it can further aid interpretation by helping readers determine whether score differences across respondents, time, or conditions are likely to reflect meaningful change rather than measurement variability (Harvill, 1991).

In contrast, when measures are developed for a specific study or clinical context and have not been subjected to systematic psychometric evaluation, clearly identifying them as author‐developed or study‐specific instruments enhances transparency. In these cases, authors could also describe the development process including the construct(s) intended to be targeted by the measure, the rationale for item selection, and the aspects of social validity intended to be assessed (e.g., treatment acceptability, perceived effectiveness, satisfaction with outcomes). Although not included in the present review, Dowling et al. (2026) provide a recent example of this type of transparent reporting. They noted that the survey used to assess social validity was designed by the authors to evaluate the safety and acceptability of the procedures used in the study. They also explicitly stated that the reliability and validity of the survey had not yet been evaluated and that the findings should therefore be interpreted with caution. Providing this information allows readers to better evaluate construct alignment and interpret the scope and limitations of the findings. Consistently reporting whether measures are validated or author‐developed instruments may also facilitate clearer differentiation across studies and improve synthesis of social validity outcomes in the literature.

## CONCLUSION

This article first provided an overview of social validity and the purpose of its assessments in behavior‐analytic research. We then reviewed the interactive process of social validity in studies published in *JABA* (2010–2020), examining how social validity assessments informed intervention adjustments. Finally, we provided recommendations and considerations for conducting social validity assessments in both research and practice as well as strategies to increase opportunities for an interactive social validity process. Ultimately, however, these decisions should be guided by the purpose of the study and the kinds of outcomes researchers and practitioners seek to inform.

Although Wolf (1978) first emphasized social validity over 45 years ago and called for behavior analysts to incorporate it into research and practice, there is still much to be done. For example, given the complexities of assessing and enhancing social validity, continued examination of how assessment‐related variables (e.g., type, evaluator, integration of multiple sources of input) influence the utility of social validity findings may strengthen the relevance, acceptability, and application of behavior‐analytic interventions for the individuals and communities they are designed to serve. In addition, greater attention to the rigor of social validity assessment methods and the interpretability of resulting data (e.g., available psychometric evidence, such as validity and reliability) is needed to ensure that conclusions about social validity are accurate, meaningful, and informative for research and practice. However, we do not want to undermine the work that has contributed to the development of social validity in behavior analysis and related efforts in fields such as psychology, medicine, and implementation science to assess stakeholder perspectives through constructs such as acceptability, feasibility, preference, and satisfaction. Even though social validity is often viewed as a one‐time measure conducted after intervention, our review of the interactive process of social validity shows that researchers have already demonstrated that social validity assessments can be dynamic and that their results can be informative for intervention adjustments that enhance social validity. We hope our call to increase the interactive process is clear for both research and practice.

Social validity is crucial in applied behavior analysis, given our responsibility to create meaningful change in human behavior (Baer et al., 1968). Its principles extend beyond applied behavior analysis to other service domains, including dental care, speech–language pathology, occupational therapy, and elder care. If practitioners actively enhance social validity based on client and stakeholder feedback, then the continued positive effects of services is more likely. Without this, we risk not only consumer disapproval but also withdrawal from treatment, discouragement of others from participating, and complaints within communities (Schwartz & Baer, 1991).

## AUTHOR CONTRIBUTIONS

The first author contributed to conceptualization, methodology, investigation, formal analysis, project administration, writing the original draft of the manuscript, and revisions of the manuscript. The second author contributed to conceptualization, methodology, supervision, and revisions of the manuscript. The third and fourth authors contributed to investigation. The fifth and sixth authors contributed to commentary of prepublication stages.

## CONFLICT OF INTEREST STATEMENT

There is no conflict of interest to declare.

## ETHICS APPROVAL

No human or animal subjects were used to produce this article.

## Supporting information

Supporting Information A
Supporting Information B
Supporting Information C
Supporting Information D

## Data Availability

The data supporting the findings of this study are available from the corresponding author upon reasonable request. Supporting Information A includes a list of included articles and a summary of social validity timing and intervention adjustments. Supporting Information B summarizes the frequency and percentage of included studies reporting social validity assessments by timing. Supporting Information C summarizes social validity dimensions (i.e., goals, procedures, and outcomes) adjusted across assessment timings. Supporting Information D summarizes whether social validity assessments across intervention timings were reported in a designated “Social Validity” section.

## Footnotes

^1^
Alternative terms for social validity used in the search were the following: social validation, social significance, social importance, socially significant, socially important, treatment validity, treatment acceptability, consumer satisfaction, satisfaction survey, Likert, acceptability scale, rating scale, rating profile, rating form, acceptability, validity, satisfaction, questionnaire, survey, subjective, and interview.
