# Supplementary material for: Social validity as an interactive process
Source: J Appl Behav Anal. 2026 Aug 14;59(4):e70079. doi: 10.1002/jaba.70079 (PMC13474287; doi:10.1002/jaba.70079)
Supplement: Supplementary file 1 [file JABA-59-0-s001.docx]

**Supporting Information A**

*Timing of Social Validity Measurement and Adjustments of Interventions in Each Article*

| **Title** | **Authors** | **Year** | **Timing of social validity assessments** | | | | **Adjustments to interventions (if any)** | | |
| --- | --- | --- | --- | --- | --- | --- | --- | --- | --- |
|  |  |  | **Before** | **During** | **After** | **Unclear** | **Adjustments** | **No adjustments to dissatisfaction** | **No dissatisfaction** |
| Comparison of simultaneous prompting and no-no prompting in two-choice discrimination learning with children with autism | Leaf et al. | 2010 |  | 1 |  |  |  |  | 1 |
| An evaluation of preference for video and in vivo modeling | Geiger et al. | 2010 |  | 1 |  |  | 1 |  |  |
| Effects of video modeling on treatment integrity of behavioral interventions | DiGennaro-Reed et al. | 2010 |  |  | 1 |  |  |  | 1 |
| Using behavioral skills training to promote safe and correct staff guarding and ambulation distance of students with multiple physical disabilities | Nabeyama & Sturmey | 2010 | 1 |  | 1 |  | 1 |  | 1 |
| Behavioral coaching to improve offensive line pass-blocking skills of high school football athletes | Stokes et al. | 2010 |  |  | 1 |  | 1 |  |  |
| Internet-based contingency management to improve adherence with blood glucose testing recommendations for teens with type 1 diabetes | Raiff & Dallery | 2010 |  |  | 1 |  |  | 1 |  |
| A point contingency for homework submission in the graduate school classroom | Rehfeldt et al. | 2010 |  |  | 1 |  |  |  | 1 |
| The effects of exergaming on physical activity among inactive children in a physical education classroom | Fogel et al. | 2010 |  |  | 1 |  |  |  | 1 |

| **Title** | **Authors** | **Year** | **Timing of social validity assessments** | | | | **Adjustments to interventions (if any)** | | |
| --- | --- | --- | --- | --- | --- | --- | --- | --- | --- |
|  |  |  | **Before** | **During** | **After** | **Unclear** | **Adjustments** | **No adjustments to dissatisfaction** | **No dissatisfaction** |
| Matrix training of pre-literacy skills with preschoolers with autism | Axe & Sainato | 2010 |  |  | 1 |  |  | 1 |  |
| Response interruption and redirection for vocal stereotypy in children with autism: A systematic replication | Cassella et al. | 2011 |  |  |  | 1 |  | 1 |  |
| Check-in/check-out: A systematic evaluation and component analysis | Campbell & Anderson | 2011 |  | 1 | 1 |  |  |  | 2 |
| Assessing the social acceptability of the functional analysis of problem behavior | Langthorne & McGill | 2011 |  |  | 1 |  |  |  | 1 |
| Transportability of equivalence-based programmed instruction: Efficacy and efficiency in a college classroom | Fienup & Critchfield | 2011 |  |  | 1 |  |  | 1 |  |
| Using differential reinforcement of low rates to reduce children's request for teacher attention | Austin & Bevan | 2011 |  |  | 1 |  |  |  | 1 |
| Measuring and reducing college students' procrastination | Perrin et al. | 2011 |  |  | 1 |  |  |  | 1 |
| Effects of general-case training, instructions, rehearsal, and feedback on the reduction of sight-reading errors by competent musicians | Dib & Sturmey | 2011 |  |  | 1 |  |  |  | 1 |
| An evaluation of the Good Behavior Game in kindergarten classrooms | Donaldson et al. | 2011 |  |  | 1 |  |  |  | 1 |
| Effects of direct instruction on the acquisition of prepositions by students with intellectual disabilities | Hicks et al. | 2011 |  |  |  | 1 |  |  | 1 |
| Establishing verbal repertoires in children with autism using function-based video modeling | Plavnick & Ferreri | 2011 |  |  |  | 1 |  |  | 1 |
| Comparison of a stimulus equivalence protocol and traditional lecture for teaching single-subject designs | Lovett et al. | 2011 |  |  | 1 |  |  | 1 |  |

| **Title** | **Authors** | **Year** | **Timing of social validity assessments** | | | | **Adjustments to interventions (if any)** | | |
| --- | --- | --- | --- | --- | --- | --- | --- | --- | --- |
|  |  |  | **Before** | **During** | **After** | **Unclear** | **Adjustments** | **No adjustments to dissatisfaction** | **No dissatisfaction** |
| Manipulation of motivating operations and use of a script-fading procedure to teach mands for location to children with language delays. | Howett et al. | 2011 |  |  |  | 1 |  |  | 1 |
| Adherence with universal precautions after immediate, personalized performance feedback. | Luke & Alavosius | 2011 |  |  | 1 |  |  |  | 1 |
| Evaluation of a self-instruction package for conducting stimulus preference assessments | Graff & Karsten | 2012 |  |  | 1 |  |  | 1 |  |
| Comparison of upright and flipped spoon presentations to guide treatment of food refusal | Sharp et al. | 2012 | 1 |  | 1 |  | 1 |  | 1 |
| Effects of three types of noncontingent auditory stimulation on vocal stereotypy in children with autism | Saylor et al. | 2012 |  |  | 1 |  |  |  | 1 |
| Effects of behavioral skills training on parental treatment of children’s food selectivity | Seiverling et al. | 2012 |  |  | 1 |  |  |  | 1 |
| An experimental analysis of the effectiveness and sustainability of a Chinese tutoring package | Wu & Miller | 2012 |  |  |  | 1 |  |  | 1 |
| The effects of matched stimulation and response interruption and redirection on vocal stereotypy | Love et al. | 2012 |  |  | 1 |  |  |  | 1 |
| A comparison of differential reinforcement and noncontingent reinforcement to treat food selectivity in a child with autism | Allison et al. | 2012 |  |  | 1 |  | 1 |  |  |
| Effects of Responding to a Name and Group Call on Preschoolers' Compliance | Beaulieu et al. | 2012 |  |  |  | 1 |  |  | 1 |
| Effects of and preference for pay for performance: An analogue analysis | Long et al. | 2012 |  | 1 |  |  |  |  | 1 |

| **Title** | **Authors** | **Year** | **Timing of social validity assessments** | | | | **Adjustments to interventions (if any)** | | |
| --- | --- | --- | --- | --- | --- | --- | --- | --- | --- |
|  |  |  | **Before** | **During** | **After** | **Unclear** | **Adjustments** | **No adjustments to dissatisfaction** | **No dissatisfaction** |
| An individualized and comprehensive approach to treating sleep problems in young children | Jin et al. | 2013 | 1 |  | 1 |  | 1 |  | 1 |
| Prevention of problem behavior by teaching functional communication and self-control skills to preschoolers | Luczynski & Hanley | 2013 |  |  | 1 |  |  |  | 1 |
| Performance pay improves engagement, progress, and satisfaction in computer-based job skills training of low-income adults | Koffarnus et al. | 2013 |  | 1 | 1 |  | 1 |  | 1 |
| Treating stereotypy in adolescents diagnosed with autism by refining the tactic of 'using stereotypy as reinforcement' | Potter et al. | 2013 |  | 1 | 1 |  | 1 |  | 1 |
| On the efficacy of a computer-based program to teach visual braille reading | Scheithauer et al. | 2013 |  |  | 1 |  |  |  | 1 |
| Training adults with an autism spectrum disorder to conduct discrete-trial training for young children with autism: A pilot study | Lerman et al. | 2013 |  |  | 1 |  |  | 1 |  |
| Teaching young adults with disabilities to respond appropriately to lures from strangers | Fisher et al. | 2013 |  |  | 1 |  |  |  | 1 |
| Effects of peer mediation on preschoolers' compliance and compliance precursors | Beaulieu et al. | 2013 |  |  |  | 1 |  |  | 1 |
| Increasing responding to others’ joint attention directives using circumscribed interests | Kryzak et al. | 2013 |  |  |  | 1 |  | 1 |  |
| Effectiveness of using noncontingent escape for general behavior management in a pediatric dental clinic | Allen & Wallace | 2013 |  |  |  | 1 |  |  | 1 |
| Efficacy of the stranger safety abduction‐prevention program and parent‐conducted in situ training | Miltenberger et al. | 2013 |  |  | 1 |  |  |  | 1 |

| **Title** | **Authors** | **Year** | **Timing of social validity assessments** | | | | **Adjustments to interventions (if any)** | | |
| --- | --- | --- | --- | --- | --- | --- | --- | --- | --- |
|  |  |  | **Before** | **During** | **After** | **Unclear** | **Adjustments** | **No adjustments to dissatisfaction** | **No dissatisfaction** |
| ^a^Internet-based contingency management increases walking in sedentary adults | Kurti & Dallery | 2013 |  |  | 1 |  |  |  | 1 |
| Producing meaningful improvements in problem behavior of children with autism via synthesized analyses and treatments | Hanley et al. | 2014 |  |  | 1 |  |  |  | 1 |
| Using goal setting and feedback to increase weekly running distance | Wack et al. | 2014 |  |  | 1 |  |  |  | 1 |
| Using audio script fading and multiple-exemplar training to increase vocal interactions in children with autism | Garcia-Albea et al. | 2014 | 1 |  | 1 |  | 1 |  | 1 |
| Training shelter volunteers to teach dog compliance | Howard & DiGennaro Reed | 2014 |  |  | 1 |  |  |  | 1 |
| Generalization of negatively reinforced mands in children with autism | Groskreutz et al. | 2014 |  |  | 1 |  |  | 1 |  |
| Effects of a classwide teacher-implemented program to promote preschooler compliance | Beaulieu & Hanley | 2014 |  |  | 1 |  |  |  | 1 |
| The effect of equipment proximity on safe performance in a manufacturing setting | Abellon & Wilder | 2014 |  |  |  | 1 |  |  | 1 |
| An evaluation of interactive computer training to teach instructors to implement discrete trials with children with autism | Pollard et al. | 2014 |  |  | 1 |  |  | 1 |  |
| Sustained effects of a visual prompt on dish storage in a hospital unit | Rubio & Sigurdsson | 2014 |  | 1 |  |  |  | 1 |  |
| The use of behavioral skills training and in situ feedback to protect children with autism from abduction lures | Gunby & Rapp | 2014 |  |  |  | 1 |  | 1 |  |
| Using tagteach to improve the proficiency of dance movements | Quinn et al. | 2015 | 1 |  | 1 |  | 1 |  | 1 |
| **Title** | **Authors** | **Year** | **Timing of social validity assessments** | | | | **Adjustments to interventions (if any)** | | |
|  |  |  | **Before** | **During** | **After** | **Unclear** | **Adjustments** | **No adjustments to dissatisfaction** | **No dissatisfaction** |
| Adults with autism spectrum disorder as behavior technicians for young children with autism: Outcomes of a behavioral skills training program | Lerman et al. | 2015 |  |  | 1 |  |  | 1 |  |
| Validating trial-based functional analyses in mainstream primary school classrooms | Austin et al. | 2015 |  |  | 1 |  |  |  | 1 |
| A comparison of two approaches to training visual analysis of AB graphs | Wolfe & Slocum | 2015 |  |  |  | 1 |  |  | 1 |
| Decreasing transition times in elementary school classrooms: Using computer-assisted instruction to automate intervention components | Hine et al. | 2015 |  |  | 1 |  |  |  | 1 |
| Teaching children with autism to respond to conversation partners’ interest | Peters & Thompson | 2015 | 1 |  |  | 1 | 1 | 1 |  |
| A comparison of video modeling, text-based instruction, and no instruction for creating multiple baseline graphs in Microsoft Excel | Tyner & Fienup | 2015 |  |  | 1 |  |  |  | 1 |
| Teaching discriminated social approaches to individuals with Angelman syndrome | Fichtner & Tiger | 2015 |  |  | 1 |  |  |  | 1 |
| Teaching statistical variability with equivalence-based instruction | Albright et al. | 2015 |  |  | 1 |  |  |  | 1 |
| The effects of video self-evaluation on skill acquisition with yoga postures | Downs et al. | 2015 | 1 |  |  | 1 | 1 |  | 1 |
| Using token reinforcement to increase walking for adults with intellectual disabilities | Krentz et al. | 2016 |  |  | 1 |  |  |  | 1 |
| Evaluation of increasing antecedent specificity in goal statements on adherence to positive  behavior-management strategies | Cohrs et al. | 2016 |  |  | 1 |  |  |  | 1 |

| **Title** | **Authors** | **Year** | **Timing of social validity assessments** | | | | **Adjustments to interventions (if any)** | | |
| --- | --- | --- | --- | --- | --- | --- | --- | --- | --- |
|  |  |  | **Before** | **During** | **After** | **Unclear** | **Adjustments** | **No adjustments to dissatisfaction** | **No dissatisfaction** |
| Effects of an interdependent group contingency on engagement in physical education | Hirsch et al. | 2016 |  |  | 1 |  |  | 1 |  |
| Using video feedback to improve horseback‐riding skills | Kelley & Miltenberger | 2016 |  |  |  | 1 |  |  | 1 |
| Evaluating a tablet application and differential reinforcement to increase eye contact in children with autism | Jeffries et al. | 2016 |  |  |  | 1 |  |  | 1 |
| Using habit reversal to decrease filled pauses in public speaking | Mancuso & Miltenberger | 2016 | 1 |  | 1 |  |  | 1 | 1 |
| Caregiver preference for reinforcement-based interventions for problem behavior maintained by positive reinforcement | Gabor et al. | 2016 | 1 |  | 1 |  |  |  | 2 |
| Efficacy of and preference for reinforcement and response cost in token economies | Jowett Hirst et al. | 2016 |  | 1 |  |  |  |  | 1 |
| Teaching social play skills to adults and children with autism as an approach to building rapport | Shireman et al. | 2016 |  |  | 1 |  |  |  | 1 |
| Examination of efficacious, efficient, and socially valid error-correction procedures to teach sight words and prepositions to children with autism spectrum disorder | Kodak et al. | 2016 |  | 1 |  |  |  |  | 1 |
| A translational evaluation of transitions | Jessel et al. | 2016 |  |  | 1 |  |  |  | 1 |
| Graduated guidance delivered by parents to teach yoga to children with developmental delays | Gruber & Poulson | 2016 |  |  | 1 |  |  |  | 1 |
| Decreasing bouts of prolonged sitting among office workers | Green et al. | 2016 |  |  | 1 |  |  |  | 1 |

| **Title** | **Authors** | **Year** | **Timing of social validity assessments** | | | | **Adjustments to interventions (if any)** | | |
| --- | --- | --- | --- | --- | --- | --- | --- | --- | --- |
|  |  |  | **Before** | **During** | **After** | **Unclear** | **Adjustments** | **No adjustments to dissatisfaction** | **No dissatisfaction** |
| Using awareness training to decrease nervous habits during public speaking | Spieler & Miltenberger | 2017 |  |  | 1 |  |  | 1 |  |
| Comparing fixed-amount and progressive-amount DRO Schedules for tic suppression in youth with chronic tic disorders | Capriotti et al. | 2017 |  |  | 1 |  |  |  | 1 |
| Programming a randomized dependent group contingency and common stimuli to promote durable behavior change | Cariveau & Kodak | 2017 |  |  | 1 |  |  |  | 1 |
| Internet based self-tailored deposit contracts to promote smoking reduction and abstinence | Jarvis & Dallery | 2017 |  |  |  | 1 |  | 1 |  |
| Evaluation of a telehealth training package to remotely train staff to conduct a preference assessment | Higgins et al. | 2017 |  |  | 1 |  |  |  | 1 |
| A microswitch-based program for promoting initial ambulation responses: An evaluation with two girls with multiple disabilities | Stasolla et al. | 2017 |  |  |  | 1 |  |  | 1 |
| Training kindergarten students lockdown drill procedures using behavioral skills training | Dickson & Vargo | 2017 | 1 |  | 1 |  | 1 |  | 1 |
| Toward meaningful outcomes in teaching conversation and greeting skills with individuals with autism spectrum disorder | Hood et al. | 2017 |  |  |  | 1 |  |  | 1 |
| Improving the interview skills of college students using behavioral skills training | Stocco et al. | 2017 |  |  | 1 |  |  |  | 1 |
| An evaluation of interdependent and independent group contingencies during the good behavior game | Groves & Austin | 2017 |  | 1 | 1 |  | 1 |  | 1 |
| Evaluation of interactive computerized training to teach parents to implement photographic activity schedules with children with autism spectrum disorder | Gerencser et al. | 2017 |  |  | 1 |  |  | 1 |  |

| **Title** | **Authors** | | **Year** | **Timing of social validity assessments** | | | | **Adjustments to interventions (if any)** | | |
| --- | --- | --- | --- | --- | --- | --- | --- | --- | --- | --- |
|  |  |  |  | **Before** | **During** | **After** | **Unclear** | **Adjustments** | **No adjustments to dissatisfaction** | **No dissatisfaction** |
| Intercontinental telehealth coaching of therapists to improve verbalizations by children with autism | Barkaia et al. | | 2017 |  |  | 1 |  |  |  | 1 |
| Teaching conversational speech to children with autism spectrum disorder using text-message prompting | Grosberg & Charlop | | 2017 | 1 |  | 1 |  | 1 |  | 1 |
| Parent-implemented behavioral skills training of social skills | Dogan et al. | | 2017 | 1 |  | 1 |  | 1 |  | 1 |
| Evaluating behavioral skills training to teach safe tackling skills to youth football players | Tai & Miltenberger | | 2017 |  |  | 1 |  |  |  | 1 |
| Step it up! Using the good behavior game to increase physical activity with elementary school students at recess | Galbraith & Normand | | 2017 |  |  | 1 |  |  |  | 1 |
| Effects of computer-based training on procedural modifications to standard functional analyses | Schnell et al. | | 2018 | 1 |  | 1 |  | 1 |  | 1 |
| Feasibility of and teacher preference for student-led implementation of the good behavior game in early elementary classrooms | Donaldson et al. | | 2018 |  | 1 | 1 |  | 1 |  | 1 |
| Achieving socially significant reductions in problem behavior following the interview-informed synthesized contingency analysis: A summary of 25 outpatient applications | Jessel et al. | | 2018 | 1 |  | 1 |  | 1 |  | 1 |
| Evaluating the Performance Diagnostic Checklist-Human Services to assess incorrect error-correction procedures by preschool paraprofessionals | Bowe & Sellers | | 2018 |  |  | 1 |  |  |  | 1 |
| Teaching help-seeking when lost to individuals with autism spectrum disorder | Carlile et al. | | 2018 | 1 |  | 1 |  | 1 |  | 1 |
| Live training versus e-learning to teach implementation of listener response programs | Geiger et al. | | 2018 |  |  | 1 | 1 |  |  | 1 |
| **Title** | **Authors** | | **Year** | **Timing of social validity assessments** | | | | **Adjustments to interventions (if any)** | | |
|  |  |  |  | **Before** | **During** | **After** | **Unclear** | **Adjustments** | **No adjustments to dissatisfaction** | **No dissatisfaction** |
| Effects of the Good Behavior Game on student and teacher behavior in an alternative school | | Rubow et al. | 2018 |  |  | 1 |  |  |  | 1 |
| Telehealth and in-person training outcomes for novice discrete trial training therapists | | Sump et al. | 2018 |  |  | 1 |  |  | 1 |  |
| Teaching children with autism spectrum disorder to tact olfactory stimuli | | Dass et al. | 2018 | 1 |  |  | 1 | 1 | 1 |  |
| Teaching social perception skills to adolescents with autism and intellectual disabilities using video-based group instruction | | Stauch et al. | 2018 | 1 |  | 1 |  | 1 | 1 |  |
| The effects of noncontingent music and response interruption and redirection on vocal stereotypy | | Gibbs et al. | 2018 |  |  | 1 |  |  | 1 |  |
| Teaching behavior technicians to create publication-quality, single-case design graphs in graphpad prism 7 | | Mitteer et al. | 2018 | 1 |  |  | 1 | 1 |  | 1 |
| Does the Good Behavior Game evoke negative peer pressure? Analyses in primary and secondary classrooms | | Groves & Austin | 2019 |  |  | 1 |  |  | 1 |  |
| Response to bullying (RTB): Behavioral skills and in situ training for individuals diagnosed with intellectual disabilities | | Stannis et al. | 2019 |  |  | 1 |  |  |  | 1 |
| An interest-based intervention package to increase peer social interaction in young children with autism spectrum disorder | | Watkins et al. | 2019 | 1 | 1 | 1 |  | 1 |  | 2 |
| Comparison of prompting procedures to teach internet skills to older adults | | Pachis & Zonneveld | 2019 |  |  | 1 |  |  |  | 1 |
| An evaluation of a punisher assessment for decreasing automatically reinforced problem behavior | | Verriden & Roscoe | 2019 | 1 |  | 1 |  | 1 | 1 |  |

| **Title** | | **Authors** | **Year** | **Timing of social validity assessments** | | | | **Adjustments to interventions (if any)** | | |
| --- | --- | --- | --- | --- | --- | --- | --- | --- | --- | --- |
|  |  |  |  | **Before** | **During** | **After** | **Unclear** | **Adjustments** | **No adjustments to dissatisfaction** | **No dissatisfaction** |
| Assessing the generality and durability of interview-informed functional analyses and treatment | | Rose & Beaulieu | 2019 |  |  | 1 |  |  |  | 1 |
| Evaluation of the accuracy, reliability, efficiency, and acceptability of fast forwarding to score problem behavior | | Machado et al. | 2019 |  |  | 1 |  |  | 1 |  |
| Reducing adolescent cell phone usage using an interdependent group contingency | | Jones et al. | 2019 |  |  | 1 |  |  | 1 |  |
| An intervention using morphology to derive word meanings for English language learners | | Davidson & O’Connor | 2019 |  |  | 1 |  |  |  | 1 |
| Evaluating small-scale simulation for training firearm safety skills | | Maxfield et al. | 2019 |  |  | 1 |  |  |  | 1 |
| Evaluating motion detection to score sleep disturbance for children: A translational approach to developing a measurement system | | Lesser et al. | 2019 | 1 |  | 1 |  |  |  | 2 |
| Awareness training reduces college students’ speech disfluencies in public speaking | | Montes et al. | 2019 | 1 |  | 1 |  |  | 1 | 1 |
| Using stimulus preference assessments to identify preferred break environments | | Castelluccio & Johnson | 2019 |  |  | 1 |  |  |  | 1 |
| ^a^Comparing self-directed methods for training staff to create graphs using Graphpad Prism | | Berkman et al. | 2019 | 1 | 1 | 1 |  | 1 | 1 | 1 |
| ^a^Using the Performance Diagnostic Checklist–Human Services to identify an indicated intervention to decrease employee tardiness | | Merritt et al. | 2019 |  |  |  | 1 |  | 1 |  |
| ^a^Technology-based contingency management and e-cigarettes during the initial weeks of a smoking quit attempt | | Martner & Dallery | 2019 |  |  |  | 1 |  | 1 |  |
| ^a^Effectiveness and acceptability of parent training via telehealth among families around the world | | Tsami et al. | 2019 |  |  | 1 |  |  | 1 |  |
|  | |  |  |  |  |  |  |  |  |  |
| **Title** | **Authors** | | **Year** | **Timing of social validity assessments** | | | | **Adjustments to interventions (if any)** | | |
|  |  |  |  | **Before** | **During** | **After** | **Unclear** | **Adjustments** | **No adjustments to dissatisfaction** | **No dissatisfaction** |
| A comparison of methods to teach foreign-language targets to young children | Matter et al. | | 2020 |  |  | 1 |  |  |  | 1 |
| A comparison of song-lyric and prose self-instruction procedures for increasing novel skills in children | Bloom et al. | | 2020 |  | 1 |  |  |  |  | 1 |
| An evaluation of parents as behavior change agents in the Preschool Life Skills program | Gunning et al. | | 2020 | 1 | 1 | 1 |  | 2 |  | 1 |
| Evaluation of a finger prompt variation in the treatment of pediatric feeding disorders | Rubio et al. | | 2020 |  |  | 1 |  |  | 1 |  |
| Improving accepting and giving compliments with individuals with developmental disabilities | Hood et al. | | 2020 | 1 |  |  | 1 |  | 1 | 1 |
| Evaluation of a telehealth parent training program in teaching self-care skills to children with autism | Boutain et al. | | 2020 | 1 |  | 1 |  | 1 |  | 1 |
| Using telehealth to teach valued skills to adults with intellectual and developmental disabilities | Pellegrino & DiGennaro Reed | | 2020 |  |  | 1 |  |  |  | 1 |
| Back to sleep: Teaching adults to arrange safe infant sleep environments | Carrow et al. | | 2020 |  |  | 1 |  |  |  | 1 |
| The effects of paired kinesthetic movements on literacy skills acquisition with preschoolers | Lozy et al. | | 2020 |  | 1 |  |  | 1 |  |  |
| Evaluating adult interaction during the Step It UP! Game to increase physical activity in children | Nieto & Wiskow | | 2020 |  |  | 1 |  |  |  | 1 |
| Comparing the effects of varied and constant preferred items on improving tummy time for typically developing infants | Morea & Jessel | | 2020 | 1 | 1 | 1 |  | 1 | 1 | 1 |
| A comparison of picture and GIF-based preference assessments for social interaction | Morris & Vollmer | | 2020 |  | 1 |  |  |  |  | 1 |

| **Title** | **Authors** | **Year** | **Timing of social validity assessments** | | | | **Adjustments to interventions (if any)** | | |
| --- | --- | --- | --- | --- | --- | --- | --- | --- | --- |
|  |  |  | **Before** | **During** | **After** | **Unclear** | **Adjustments** | **No adjustments to dissatisfaction** | **No dissatisfaction** |
| An evaluation of matrix training to teach college students piano notes and rhythms | Langton et al. | 2020 |  |  | 1 |  |  |  | 1 |
| Utilizing in-vivo feedback for caregiver training of pediatric feeding protocols | Bachmeyer-Lee et al. | 2020 |  |  | 1 |  |  | 1 |  |
| An evaluation of synchronous reinforcement for increasing on-task behavior in preschool children | Diaz de Villegas et al. | 2020 |  | 1 |  |  | 1 |  |  |
| Caregivers can implement play-based instruction without disrupting child preference | Pisman & Luczynski | 2020 |  |  |  | 1 |  |  | 1 |
| A randomized clinical trial of a virtual-training program for teaching applied-behavior-analysis skills to parents of children with autism spectrum disorder | Fisher et al. | 2020 |  |  | 1 |  |  |  | 1 |
| Self-guided behavioral skills training: A public health approach to promoting nurturing care environments | Shea et al. | 2020 |  |  | 1 | 1 |  | 2 |  |
| Technology-based versus in-person deposit contract treatments for promoting physical activity | Stedman‐Falls & Dallery | 2020 | 1 |  | 1 |  |  |  | 2 |
| Safe to sleep: Community-based caregiver training | Vladescu et al. | 2020 |  |  | 1 |  |  |  | 1 |
| Tummy time without the tears: The impact of parent positioning and play | Mendres‐Smith et al. | 2020 | 1 | 1 | 1 |  | 2 |  | 1 |
| Achieving socially significant reductions in problem behavior following the interview-informed synthesized contingency analysis: A summary of 25 outpatient applications | Jessel et al. | 2020 |  |  | 1 |  |  |  | 1 |
| Stimulus presentation versus stimulus removal in the Good Behavior Game | Silva & Wiskow | 2020 |  | 1 | 1 |  |  | 1 | 1 |

| **Title** | **Authors** | **Year** | **Timing of social validity assessments** | | | | **Adjustments to interventions (if any)** | | |
| --- | --- | --- | --- | --- | --- | --- | --- | --- | --- |
|  |  |  | **Before** | **During** | **After** | **Unclear** | **Adjustments** | **No adjustments to dissatisfaction** | **No dissatisfaction** |
| Increasing accuracy of rock-climbing techniques in novice athletes using expert modeling and video feedback | Walker et al. | 2020 |  |  | 1 |  |  |  | 1 |
| Using auditory feedback in body weight training | Vorbeck & Bördlein | 2020 | 1 |  | 1 |  |  | 1 | 1 |
| A comparison of accumulated and distributed reinforcement periods with children exhibiting escape-maintained problem behavior | Fulton et al. | 2020 |  | 1 |  |  |  |  | 1 |
| A comparison of response interruption and redirection and competing items on vocal stereotypy and appropriate vocalizations | Shawler et al. | 2020 |  |  | 1 |  |  |  | 1 |
| An evaluation of parent preference for prompting procedures | Halbur et al. | 2020 | 1 | 1 | 1 |  | 1 |  | 2 |
| Assessment and treatment of high-risk challenging behavior of adolescents with autism in an aquatic setting | Dowdy & Tincani | 2020 |  |  | 1 |  |  |  | 1 |
| Comparison of two TAGteach error-correction procedures to teach beginner yoga poses to adults | Ennett et al. | 2020 |  |  |  | 1 |  |  | 1 |
| Effect of conducting behavioral observations and ratings on staff implementation of a paired-stimulus preference assessment | Marano et al. | 2020 |  |  | 1 |  |  |  | 1 |
| Effects of a problem‐solving strategy on the independent completion of vocational tasks by adolescents with autism spectrum disorder | Lora et al. | 2020 |  |  |  | 1 |  |  | 1 |
| Effects of exposure to prompts on the acquisition of intraverbals in children with autism spectrum disorder | Kay et al. | 2020 |  |  |  | 1 |  |  | 1 |
| Efficacy and social validity of procedures for improving conversational skills of college students with autism | Mann & Karsten | 2020 | 1 |  | 1 | 1 | 1 |  | 2 |
|  |  |  |  |  |  |  |  |  |  |
| **Title** | **Authors** | **Year** | **Timing of social validity assessments** | | | | **Adjustments to interventions (if any)** | | |
|  |  |  | **Before** | **During** | **After** | **Unclear** | **Adjustments** | **No adjustments to dissatisfaction** | **No dissatisfaction** |
| Efficacy of teacher-implemented good behavior game despite low treatment integrity | Joslyn & Vollmer | 2020 |  |  | 1 |  |  | 1 |  |
| Enhancing the training integrity of human service staff using pyramidal behavioral skills training | Erath et al. | 2020 |  |  | 1 |  |  |  | 1 |
| Life skills instruction for children with developmental disabilities | Robison et al. | 2020 |  |  |  | 1 |  |  | 1 |
| Reducing excessive questions in adults at adult-day training centers using differential-reinforcement-of-low rates | Otalvaro et al. | 2020 |  |  | 1 |  |  |  | 1 |
| Reducing risk of head injury in youth soccer: An extension of behavioral skills training for heading | Quintero et al. | 2020 | 1 |  |  | 1 | 1 |  | 1 |
| Reducing speech disfluencies during public speaking using brief habit reversal | Pawlik & Perrin | 2020 |  |  | 1 |  |  | 1 |  |
| Teaching caregivers to implement mand training using speech generating devices | Suberman & Cividini-Motta | 2020 | 1 |  | 1 |  |  |  | 2 |
| Teaching caregivers to respond safely during agitated states before aggression using simulation training | Metoyer et al. | 2020 |  |  | 1 |  |  |  | 1 |
| Using the Step it UP! Game to increase physical activity during physical-education classes | Normand & Burji | 2020 |  |  | 1 |  |  |  | 1 |
| Using video modeling to teach play comments to dyads with ASD | Ezzeddine et al. | 2020 | 1 |  |  | 1 | 1 |  | 1 |

*Note*. Titles with asterisks (^a^) indicate articles that were included in our review but not Leif et al. (2024). Specifically, those five articles were: Kurti & Dallery (2013); Berkman et al. (2019); Merritt et al. (2019); Martner & Dallery (2019); Tsami et al. (2019). Values in columns indicate the presence or number of the specified characteristic (e.g., 1 = one social validity assessment reported; 2 = two assessments); blank cells indicate not reported.

**Supporting Information B**

*Timing of Social Validity Assessments Reported*

| **Timing of social validity assessments** | **Total** | **Percentage** |
| --- | --- | --- |
| Before | 36 | 21.81% |
| During | 24 | 14.54% |
| After | 123 | 74.54% |
| Unclear | 34 | 20.60% |

*Note*. This table summarizes the frequency and percentage of included studies reporting social validity assessments by timing (before, during, or after intervention, or unclear). Percentages were calculated based on the total number of articles included in the review (N = 165).

**Supporting Information C**

*Social Validity Dimensions of Adjustments Across Timings*

|  | **Goals** | | **Procedures** | | **Outcomes** | |
| --- | --- | --- | --- | --- | --- | --- |
| **Timing of social validity assessments** | **Total** | **Percentage** | **Total** | **Percentage** | **Total** | **Percentage** |
| Before | 11 | 42.31% | 11 | 42.31% | 11 | 42.31% |
| During | 0 | 0% | 12 | 100% | 0 | 0% |
| After | 0 | 0% | 0 | 0% | 0 | 0% |
| Unclear | 0 | 0% | 0 | 0% | 0 | 0% |

*Note*. Of the studies that made adjustments, this table summarizes the social validity dimensions (i.e., goals, procedures, outcomes) that were adjusted across different assessment timings. Percentages were calculated based on the total number of adjustments made within each intervention timing category: before (n = 26), during (n = 12), after (n = 0), and unclear (n = 0).

**Supporting Information D**

*Social Validity Description Under a Specified Social Validity Section*

| **Timing of social validity assessments** | **Total** | **Percentage** |
| --- | --- | --- |
| Before | 13 | 36.11% |
| During | 2 | 8.33% |
| After | 84 | 68.29% |
| Unclear | 24 | 70.59% |

*Note.* This table summarizes whether social validity assessments across intervention timings (i.e., before, during, or after intervention, or unclear) were described in a specified “Social Validity” section. Percentages were calculated based on the total number of assessments within each intervention timing category: before (n = 36), during (n = 24), after (n = 123), and unclear (n = 34).
